# What Environmental Metrics Are Used in Scientific Research to Estimate the Impact of Human Diets?

**DOI:** 10.3390/nu16183166

**Published:** 2024-09-19

**Authors:** Magaly Aceves-Martins, Anneli Lofstedt, Naara Libertad Godina Flores, Danielle Michelle Ortiz Hernández, Baukje de Roos

**Affiliations:** The Rowett Institute, University of Aberdeen, Foresterhill, Aberdeen AB25 2ZD, UK; anneli.lofstedt2@abdn.ac.uk (A.L.); a01377130@exatec.tec.mx (N.L.G.F.); a01630998@exatec.tec.mx (D.M.O.H.)

**Keywords:** diet, environmental impact, metrics, data, artificial intelligence, systematic review

## Abstract

Background/Objectives: Metrics drive diagnosis, and metrics will also drive our response to the challenge of climate change. Recognising how current scientific research defines and uses metrics of the environmental impact of human diets is essential to understand which foods, food groups, or dietary patterns are associated with a higher environmental impact. Methods: This research, aided by artificial intelligence (AI), aimed to search, map, and synthesise current evidence on the commonly used definitions and metrics of the environmental impacts of human diets. Results: We identified 466 studies measuring the environmental impact of diets. Most studies were from North American or European countries (67%), with data mainly from high-income countries (81%). Most studies did not include methods to recall the provenance of the foods consumed. Most (53%) of the studies only used one metric to estimate the environmental impact of human diets, with 82% of the studies using GHGE. Conclusions: Agreement on how the environmental impact of diets is measured and more comprehensive and accurate data on the environmental impact of single foods is essential to better understand what changes in food systems are needed, at a consumer and policy level, to make a well-meaning change towards a more sustainable diet.

## 1. Introduction

The relationship between food systems and the environment is complex and bidirectional—food production and consumption have a significant impact on the environment, for example, through greenhouse gas (GHG) emissions and extensive land and water use, whereas climate change also unsettles food systems through heat and extreme weather, biodiversity loss, and ecosystem alterations [1,2]. Moreover, climate change disruption can impact socioeconomic outcomes, such as increased poverty [3], food insecurity [4], and population displacement [5], affecting dietary patterns.

Describing “sustainable diets” is challenging as the methods to do so have been described as weak, fragmented, and arbitrary [6,7]. Moreover, there are several interdisciplinary areas studying environmental impact (e.g., agriculture, nutrition, economy, policy) which include different scopes and methods [8,9]. Some reviews about the measurements of sustainable diets were produced some years ago [10,11]. Nevertheless, climate and environmental data have grown in size and complexity in the last decade. Because of the lack of standardised methodologies to measure environmental impact, defining and measuring an “environmentally sustainable diet” has become a convoluted task. For this reason, collating and synthesising available data about “environmentally sustainable diets” using traditional evidence synthesis approaches may no longer be feasible [12,13,14]. In recent years, several artificial intelligence (AI) tools have been developed to review evidence systematically. Such methods have been used previously in clinical areas [15,16,17], or to map the effect of climate change on health [12]. Such tools could also offer a practical approach to map the evidence of the measurement of the impact of diets on climate change and the evidence of climate change’s impact on diets.

In relation to the climate change crisis, sustainable food systems have become a politically important topic in recent years. We hypothesise that GHG emissions are the measurement most frequently used when estimating the impact human diets have on the environment. Considering the complexity and amount of evidence currently being produced on this topic, a systematic search of the literature, aided by AI, should be better placed to comprehensively map the metrics used in current research to measure the environmental impact of diets. Therefore, this research aims to search, map, and synthesise the most recent and complete evidence on the commonly used definitions and metrics of the environmental impacts of human diets. This will be imperative for the development, implementation, and monitoring of policies in this area.

## 2. Materials and Methods

A systematic review protocol was produced and registered in the OSF Registries [18] and was reported following the PRISMA guidelines [19]. Our research question and methods were based on the Population/problem, Interest, Context, and Time and Scope (PICoST) approach (Table 1). Our inclusion and exclusion criteria were defined as follows:

Population/problem: Any studies considering human diets or human food production, retail, or consumption, in any geographical area were included. Animal-model studies or studies that did not include data relating to human diets were excluded from this review. Also, those studies evaluating the environmental impact of specific food products (e.g., crops) without specifying if this was for human consumption were excluded.

Interest: One of the essential points of this review was the identification of environmental metrics; hence, the data sources and the type of data within each study were relevant to this review. Scientific studies measuring the impact of human diets, a combination of food groups, or dietary guidelines implementation on the environment or biodiversity loss were included. Exclusions comprised studies analysing single food groups or items. Also, studies that evaluated practices to improve agricultural processes or farming techniques were excluded.

Context: Studies measuring the impact of human diets on environmental markers during the food production (data from food grown, harvesting, or processing and cooking), retail (data from food purchase at retail points), or consumption (data from dietary recalls) phase were included in this review. Included studies were not limited to a specific life cycle assessment or stages. Studies evaluating the environmental impact of food waste, food plastic packaging, or human excretion of food products were excluded. Also, studies assessing the footprints of the overall agricultural sector or agroforestry systems, without relating to a specific diet or different food groups, were excluded. Finally, studies evaluating policies or taxation exercises that attempt to change the environmental impacts of diets were excluded.

Scope and Time: Scientific publications published in academic journals between January 2012 and November 2023 were included to focus on the contemporary study of the environmental impact of diets. Protocols, conference abstracts or posters, reviews, opinion manuscripts, trade and industry journals, book chapters, monographs, theses, technical reports, conference proceedings, or government publications were excluded.

### 2.1. Searches

Searches were conducted in EMBASE, Medline, Agricultural & Environmental Science Collection, and AGRICOLA. We designed a comprehensive data search using strings combining two key concepts: diet and climate change. To ensure no relevant term was omitted in the research, we first created a set of initial search terms from existing relevant papers, reviews, and internal team expertise. Then we linked such terms with Boolean connectors to identify pertinent references of titles or abstracts. All searches were done by using English-only search terms. The search strategy can be found in Supplementary Material S1. Original searches were done in July 2022 and updated in November 2023.

### 2.2. Screening

A large number of references was anticipated. For this reason, we used AI, specifically a supervised active learning machine-learning approach called ASReview [20], to aid in the title and abstract screening. This algorithm was trained to predict the human reviewer’s decisions across the complete list of retrieved references. Based on our inclusion and exclusion criteria, one reviewer (MA-M) manually screened a subset of relevant references to train the algorithm, which automatically predicted the most likely relevant references. The complete steps on how this AI tool was trained are provided in Supplementary Material S2. A data-driven strategy to train the algorithm was used (i.e., the reviewer will decide to stop after the algorithm retrieves x amount of consecutive irrelevant papers). Finally, three reviewers (MA-M, NLGF, and DMOH) revised a 10% random list of irrelevant references to check the consistency of the AI decision-making.

### 2.3. Data Extraction

Once the AI tool identified the relevant papers, a full-text review was conducted by three reviewers (MA-M, NLGF, and DMOH). For those papers that met our inclusion criteria, relevant data were extracted in a data extraction form designed for this review. The data extraction form included general characteristics of the study (e.g., author, year of publication, country, design, etc.); data relevant to the food chain stage (e.g., food production, transportation, preparation, or consumption); and the environmental or sustainability definition and measurements. The primary outcome of his review was the identification of the most frequently used environmental impact metrics of human diets across scientific research. Data relevant to this outcome (e.g., type of measurement, units, validity, usability) were extracted and analysed. The metrics comprised in the Product Environmental Footprint Category Rules (GHG emissions, land use, water use, resource use minerals and metals, resource use fossils, ozone depletion, human toxicity, human toxicity-non-cancer, ionising radiation-human health, photochemical ozone formation-human health, acidification, eutrophication-terrestrial, eutrophication-freshwater, eutrophication-marine, and ecotoxicity-freshwater), described by the European Union [21], were included in the data extraction form. In addition, biodiversity loss and any other metric reported in the studies were also recorded.

### 2.4. Synthesis

Our review comprised a mapping of the definitions and metrics of the environmental impact of diets, aiming to describe the nature of the data used. It did not include a critical appraisal of the study’s validity or synthesis of results, and therefore risk of bias appreciation was not assessed. Due to the heterogeneity across studies, this work included a narrative synthesis of the main characteristics that were graphed. We examined emerging patterns with the extracted data to identify any explanations for differences in metrics used across different geographical areas or academic disciplines. Graphs were built using R software (version number 4.4.1) using the libraries “networkD3” and “chorddiag”.

## 3. Results

**Figure 1:**
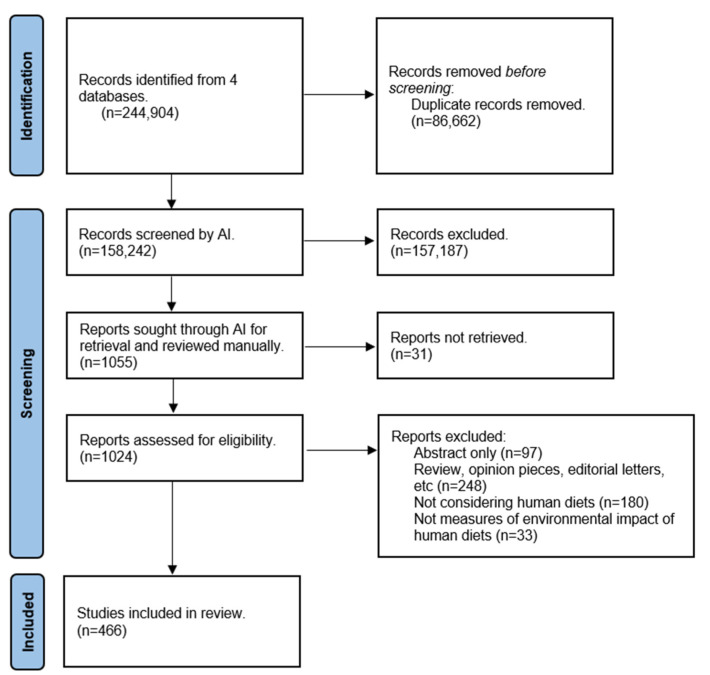
PRISMA Flowchart.

**Figure 2:**
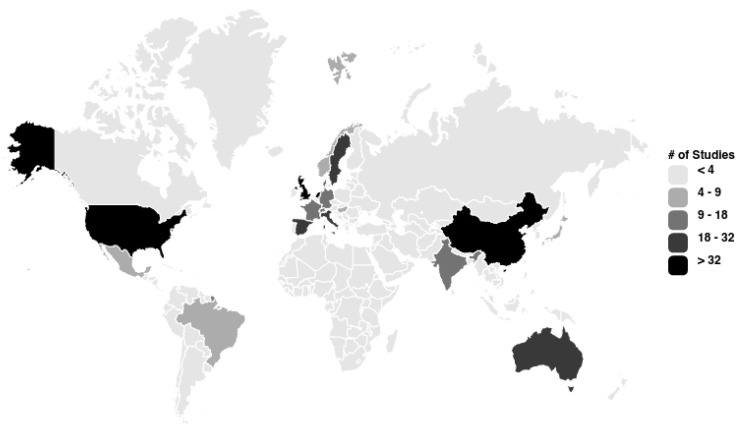
Origin of the included studies.

Our searches retrieved 158,242 single references. After training an algorithm, 1055 unique references were highlighted as relevant for our review (Figure 1). After a full-text review, 466 studies were included in this review. The complete list of included references can be seen in Supplementary Material S3. Most included studies were from North American or European countries (67%), with data mainly derived from high-income countries (81%) (Figure 2).

**Figure 3:**
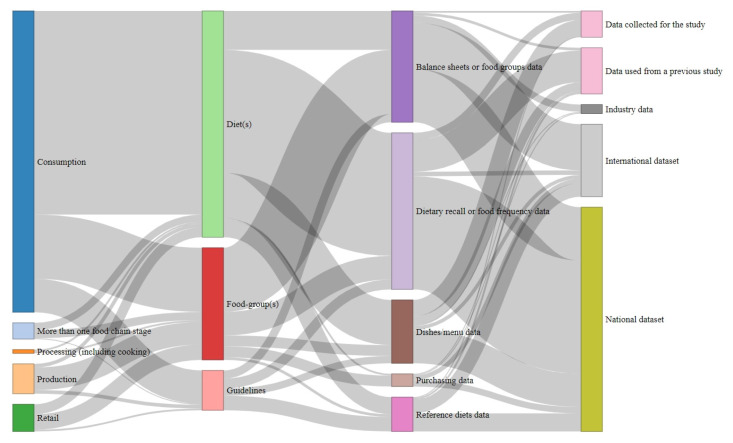
Relationship between the type of data identified.

Most of the studies (81%) evaluated the environmental impact of the consumption of diets or different food groups, while the rest evaluated the environmental impact of either the production (7%), retail (7%), mixed stages of the food chain (4%), or food processing (1%) (Figure 3). Most of the studies (61%) evaluated consumption patterns of diet(s) (e.g., traditional national diets, vegetarian, vegan, etc.), and the rest of the studies evaluated the data in light of different food groups (28%), or reference diets stipulated by guidelines (11%). The dietary data used in the studies had different origins: 44% was gathered through dietary recalls or food frequency questionnaires (e.g., 24-h dietary recall), 27% used balance sheets data, 16% evaluated data from different menus, dishes, or recipes, 10% modelled diets based on national or international guidelines, and 3% evaluated consumers purchasing data (Figure 3). Most of the studies (60%) used national datasets (e.g., the UK National Diet and Nutrition Survey), 18% used international datasets (e.g., FAO balance sheets), 13% used data from previous studies that included a dietary component, 7% collected dietary data to be analysed for environmental metrics, and 2% used data provided by industries (Figure 3; an interactive version of Figure 3 is available in Supplementary Material S4).

**Figure 4:**
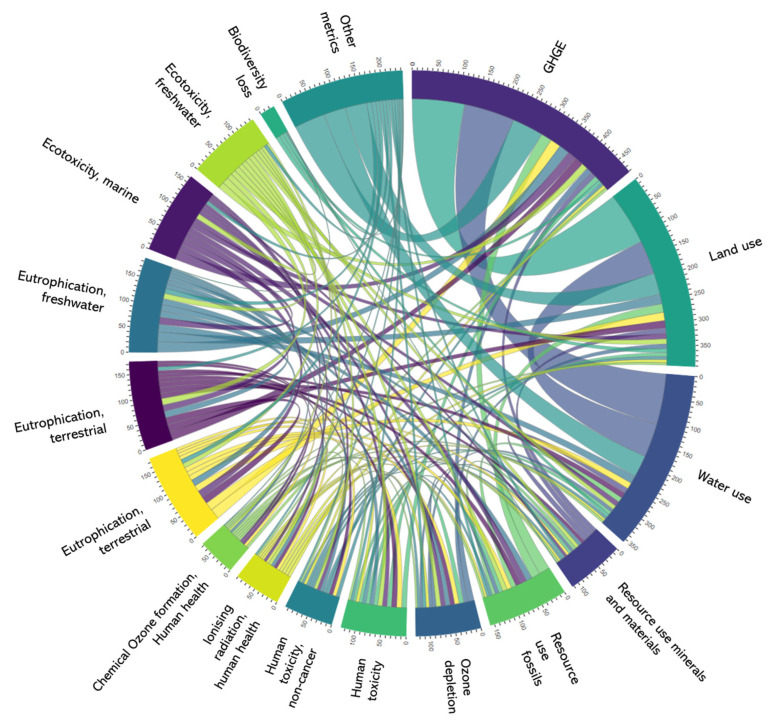
Combination of sustainability metrics when studying human diets.

The median number of environmental markers measured per study was 1.1, which reflects that most (53%) of the studies only used one metric to estimate the environmental impact of human diets. The metric most widely used was GHG emissions, with 82% of the studies using this metric and 38% of the studies using GHG emissions as a single environmental metric. Other metrics used by studies included water footprint (40%) and land use (34%). For those studies using more than one metric, the most common combination of metrics included GHG emissions and land or water use (Figure 4; an interactive version of Figure 4 is available in Supplementary Material S5).

The metrics shown in Figure 4 are aligned with the ones the EU considers relevant environmental impact metrics. However, some studies also reported other metrics (e.g., energy use), and 20 different metrics were identified overall. The environmental impact metrics were retrieved from a third-party source (e.g., published previous studies, grey literature, national reports) in the majority of studies (92%), and the rest estimated the environmental impact for the sample in the study or used commercial sources. From those studies using a third-party source, most (59%) conducted meta-revisions (in some cases systematic reviews) of papers to collate, impute, and then estimate values of the environmental impact of diets. Other studies (36%) used well-known data sets (e.g., SHARP-ID or Blonk) to impute values within their studies. Most studies reported using a life-cycle assessment (LCA) when reporting how the environmental data was used. However, how the LCA was collected, or which phases were considered within the LCA analysis, was unclear for 37% of the studies. Around 37% of the studies mentioned using the entire cycle, also called farm-to-grave data. Around 12% of the studies used farm to-gate data (production data), 6% used farm-to-retail data, and 8% used farm-to-consumption (farm-to-fork) data.

## 4. Discussion

We found a wide heterogeneity in the number and type of metrics used to define the environmental impact of diets. GHG emissions was the most frequently used metric, followed by water and land use, for evaluating the environmental impact of human diets. Most of the GHG emissions data used were imputed from open-access references or databases, but the accuracy of such data needs to be questioned based on differences in food provenance, and on applied LCA methodologies with different interpretations alongside the missing standardisation of these terms. The accurate assessment of the environmental impact of our food system and the diets we consume is essential to better understand what changes are needed, at a consumer and policy level, to make a well-meaning change. However, such assessments are challenging for various reasons.

Firstly, as indicated in this study, multiple separate metrics can be considered for the measurement of the environmental impact of our food systems, such as those described by the Product Environmental Footprint Category Rules [21]. Our review, covering the last decade of published literature, identified over 20 metric systems utilised as proxies to assess the impact our diets have on the environment. It is well known that several factors and variables collectively influence climate change, but no single integrated metric captures a combination of these outcomes. Only one or two metrics (usually GHG emissions) are mostly reported in studies, as these seem to be best understood by the general public, and future strategies involving public meal choice changes would likely use these metrics [22,23,24]. This represents a limitation, as scientific research that estimates the environmental impact of human diets on GHG emissions only may overlook other important food-based environmental impacts, such as, for example, land and water use.

Secondly, we found that most studies (92%) use imputed data, such as GHG emissions, from open-access databases. These data are usually post-processed for easy use or calculated for a specific location and/or specific practices that might not be applicable to a different context [6,25]. Environmental impact data continuously change because of improved instruments and methods to calculate such data, data processing, and changing practices [6]. Some authors have questioned the applicability of such data to study long-term environmental impact, and suggested that the robustness of LCA data needs to be revised often, as it involves an evolving food production system [26]. For example, 37% of the studies mention LCA without specifying which phases of the food chain were considered. In addition, some authors search the literature, not always systematically, to collate environmental data that is subsequently used for analysis [27,28,29], which means it will require more work to validate such studies and replicate methods for future use [30]. Moreover, we found that current evidence has been generated mostly in high-income countries (81%), primarily in North America and Europe (67%), which raises concerns about the applicability of the data in other regions. All of these factors imply that data from different origins are being mixed between methodological recollections and geographical areas. For instance, most studies from developing countries use references from developed countries, and some authors acknowledged that the data they used might be irrelevant to their context as products, processes, and consumption patterns differ [31,32,33]. But this also represents an opportunity for industries and governments to join efforts and build centralised data with robust provenance that allows academia, consumers, and industries to access valid and relevant environmental impact data for use in a specific context. That origin and quality of data do affect the outcome was shown by Sugimoto et al. 2021 [34], who compared three different ways to estimate and impute GHG emissions data from the same diet. Whilst strong correlations were observed between the three methods, the mean total diet-related GHG emissions significantly differed.

Thirdly, it is well known that collecting data on dietary intake often lacks accuracy in reporting the quantity and quality of diets at an individual and population level [35]. Our review shows that most dietary data come from national surveys that collect data through dietary recalls or food frequency questionnaires. These provide aggregated data of several participants in a standardised way. Estimating the environmental impact of foods or diets would need more detailed information on the provenance of foods, as was done by Baroni 2014 et al. [36]. In addition, most studies generally do not consider import-export fluxes, cooking techniques or times, procedures, and storage, for the foods that are consumed, and which also impact on the environment [37,38,39]. For example, studies should consider LCA approaches and account for all the environmental externalities associated with specific foods during the production process [26]. Although there are standard frameworks to estimate and report LCA [26], 37% of studies included in this review did not report on how LCA of imputed data were recorded. For the 63% of studies that did report using LCA data, not all considered the same food chain stages, with transportation or refrigeration of foods usually not accounted for. Such data would be relevant to countries like the UK, where only 7% of fruits are produced domestically, with the rest being imported, mostly (70%) from outside of Europe, and where the impacts of air-freighted fresh vegetables are around five times higher than those produced domestically [37,38].

A couple of systematic reviews on this topic have been previously published. Jones et al. 2016 [11] and Hallstrom et al. 2015 [10] looked at the assessment of different scenario analyses measuring the environmental impact of human dietary change, as well as the empirical research on sustainable diets to identify the components of sustainability. However, this research was conducted almost a decade ago, and none of the previous reviews reflected on the metrics being used as part of the research methodology. This systematic review focused on the methods applied in order to obtain and use data on diets and their environment. The amount of evidence mapped was considerably different from previous reviews, as we were able to identify 466 relevant references (compared to 113 [11] and 14 [10], in previous reviews). The strengths of this work include the systematic and comprehensive search for evidence that, as expected, detected a very large number of studies. Using AI tools to search for and review the many references helped us predict the most relevant studies for this review, and saved resources such as time. This is also a limitation, as AI tools and machine learning are relatively new methodologies for conducting systematic reviews, and such technologies are still developing [15,16]. For this reason, there is a risk of missing relevant data throughout the process.

The implications of this work for policy and practice include a call to create better and more accurate data on the environmental impact of foods, which will be relevant to research, policy, and consumers, when measuring the environmental impact of diets. From a scientific perspective, interdisciplinary approaches can integrate and align efforts that allow a better and more holistic understanding of how diets impact on the environment, and which metrics should be used. Moreover, we propose methodological improvements, such as reporting data on provenance, and such data need to be improved, transparent and accessible. From an environmental perspective, previous issues with guidelines, data, or procedures to collect environmental data have been raised. For instance, in the UK [40], national guidelines for public procurement (e.g., DEFRA) are mainly based on existing certification schemes (e.g., the FAO code of conduct for responsible fisheries) [41] or large national datasets (e.g., National Dietary and Nutrition Survey, where dietary data might be poorly reported in terms of environmental impact), and on promoting resource-efficient practices of food service operations (e.g., reduction of food and packaging waste). We need a contextualised life cycle perspective (e.g., relevant to the geographical location), as the current data might not showcase the supply chain stages causing the highest impacts [40]. Moreover, researchers, policymakers, and industry need to define a clear, relevant, and accurate framework for measuring environmental impact with clear system boundaries, including country-specific data. This will ensure the comparability of sources and data across studies and policies. Accurate data on environmental impact will also allow a more holistic approach to optimising human diets in terms of nutrition, environmental impact and food security.

## 5. Conclusions

Our review shows that a high amount of research is being produced in terms of the environmental impact of human diets. Currently, we do have insight on which foods or food groups are less environmentally friendly, mainly by using GHG emissions as an indicator. However, data on other sustainability aspects are rare and metrics incomplete. There is a global need to ensure nutrition and food security for a growing population while the climate changes. A dietary shift, especially in high-income countries with Western-type diets, is critical to achieving our climate targets [42], and improved dietary and environmental impact data, through multidisciplinary contextualisation and harmonisation of data, are essential to monitoring progress towards achieving these targets. Building comprehensive measurements able to capture the biodiversity impact, social, economic, or nutritional aspects of human diets, should be considered collectively with the environmental impact metrics. There is a need to transform and monitor food systems, but there is also a need to link planetary and human health [43]. As consumers and researchers, we need to accurately quantify the associated environmental savings or costs of the foods we consume to identify the dietary changes required to achieve a climate-friendly and healthy diet. For this, the most accurate metrics are essential.

## Figures and Tables

**Table 1:** Population/problem, Interest, Context, and Time and Scope of this review.

Item	Description
Population/problem	Studies considering human diets or human food production, retail, or consumption, in any geographical area.
Interest	Identification of metrics and definitions of the environmental impact of human diets.
Context	Climate change and the environmental impact of human diets and food consumption.
Scope and Time	Scientific publications published in academic journals between January 2012 and July 2022 were included to focus on the contemporary study of the environmental impact of diets.

## Data Availability

This research was conducted using published materials. Supplementary Materials include the full list of included papers.

## Supplementary Materials

The following supporting information can be downloaded at https://www.mdpi.com/article/10.3390/nu16183166/s1; the search strategy can be found in Supplementary Material S1. The AI training methods can be found in Supplementary Material S2. The list of revised and included papers can be found in Supplementary Material S3, and an interactive version of Figure 3 and Figure 4 is available in Supplementary Materials S4 and S5, respectively.
