# Supplementary material for: What Environmental Metrics Are Used in Scientific Research to Estimate the Impact of Human Diets?
Source: Nutrients. 2024 Sep 19;16(18):3166. doi: 10.3390/nu16183166 (PMC11435316; doi:10.3390/nu16183166)
Supplement: Supplementary file 1 [file nutrients-16-03166-s001.zip › Supplementary Material S1 Search Strategy 27.08..2024.pdf]

**Supplementary Material S1. Search Strategy**

| | |
|---|---|
| 1 | diet* or food* or nutrition* or menu* or cook* or food-level or nutritional or dietary or "Dietary Choices" or "food menu" or "food choices" or "dietary patterns" or "nutritional quality" or "dietary behaviours" or "cooking" or "food consumption" or "sustainable diet" or "food industry" or "food chain" or "food packing" or "food supply" or "food storage" or "food supply chain" or "food transportation" or "food system" or "food policy" or "Global food" or "Sustainable food production systems" or "Resource intensive foods" or "Dietary change" or "food security" or "food insecurity" or "cheaper food paradigm" or "over-consumption" or "under-consumption" or "calories" or "nutrients" or "human dietary shifts." |
| 2 | Environment* or sustainability or sustainb* or Methane or Ecosystem or Biodiversity or Eutrophication or "Climate Change" or "Environmental Monitoring" or "environmental sustainability" or "Environmental Impact" or "Global warming" or "Greenhouse Gas" or "Greenhouse Effect" or "Greenhouse gas emission" or "GHGE" or "GHG emissions" or "GHGE (greenhouse gas emissions)" or "Natural Resources" or "Carbon Dioxide" or "CO2" or "carbon dioxide equivalent" or "Carbon Footprint" or "Stratospheric ozone depletion" or "Ionizing radiation" or "Ozone formation" or "Terrestrial acidification" or "Terrestrial ecotoxicity" or "Mineral resource scarcity" or "Fossil resource scarcity" or "Freshwater eutrophication" or "Marine eutrophication" or "Marine ecotoxicity" or "Water Resource" or "Water Supply" or "Water Use" or "Water footprint" or "Water consumption" or "Blue footprint" or "Freshwater withdrawals" or "Freshwater ecotoxicity " or "Scarcity-weighted water" or "blue water footprint" or "climate impact" or "LCA" or "Life cycle assessment" or "Land Use" or "Land Use Change" or "sustainability performance" or Habitat or "Restoring ecosystems" or "land protection" or Monoculture or polyculture or "agricultural production" or "intensive farming" or "Species extinction" or "Homogenisation" or "farmland" or "Genetic diversity" or "erosion" or "Environmental footprint" or "Ecosystem resilience" or "Nature-based solutions" or "Aichi Biodiversity Targets" or "TEEBAgriFood Evaluation Framework" or Biodiversity |
| 3 | 1 and 2 |
