# Supplementary material for: What Environmental Metrics Are Used in Scientific Research to Estimate the Impact of Human Diets?
Source: Nutrients. 2024 Sep 19;16(18):3166. doi: 10.3390/nu16183166 (PMC11435316; doi:10.3390/nu16183166)

Supplementary Material 2. AI algorithm (ASReview) training:

In this systematic review, the AI tool ASReview (through ASReview Lab) was used to screen titles and abstracts. After searches were done and deduplicated, a list of single references was produced. Once this list was uploaded, ASReview was initially trained using prior knowledge of relevant references (https://doi.org/10.3390/ijerph19063191, https://doi.org/10.3389/fnut.2022.851826, https://doi.org/10.1016/S2542-5196(21)00254-0) selected based on the experience of the authors (two of which are authored by the some of the authors of this review). However, due to the large number of references retrieved for this review, after training with prior knowledge of relevant references, ASReview ranked all unlabelled references yet to be selected on eligibility. After this initial ranking, 40 relevant and 80 irrelevant were classified. So, these 40/80 references were considered prior knowledge.

This AI tool uses active learning, which some call "human-in-the-loop", to help the algorithm predict the reviewer's decisions. This tool includes different classifier models to train the machine learning algorithm so that the relevance of the references can be determined. From the classifier models available in ASReview, the default setting combination Naïve Bayes and term frequency-inverse document frequency (TF-IDF) were selected.

Afterwards, based on prior knowledge, the classifier ranked unlabelled references from highest to lowest probability of being relevant. One reviewer (MA-M) read the title and abstract of references, ranked the highest and decided to categorise the references as "relevant" or "irrelevant" following the inclusion and exclusion criteria. See **Diagram S1** for guidance on the decisions made while selecting the references. Based on the prior knowledge and the decisions made by the reviewer (MA-M), ASReview made a new ranking constantly to show that the next top-ranked reference was proposed who decided eligibility. The process of AI taking additional prior knowledge into account for each ranking and a reviewer making decisions was repeated until the predefined data-driven strategy (i.e., the reviewer will decide to stop after the algorithm retrieves x amount of consecutive irrelevant references) reached 300 subsequent irrelevant references. (**Figure S1**)

The main aim of this review was to capture those references that measure the environmental impact of human diets or food consumption. However, due to the comprehensive searches, several references considered the environmental impact of human diets, which was irrelevant to this review. For this reason, and to better guide the algorithm, reviews of any type considering the effect of diets on the environment were tagged as relevant. Still, these were excluded in the full-text assessment process.

Once the relevant references were identified by ASReview, three reviewers (MA-M, NLGF, MD) revised the full text to assess eligibility. Also, these same three reviewers revised 100 references, randomly selected, and categorised as irrelevant to cross-check accuracy and consistency. Any disagreement regarding inclusion was discussed across all the authors of this systematic review.

Diagram S1: Decision making to train AI tool.

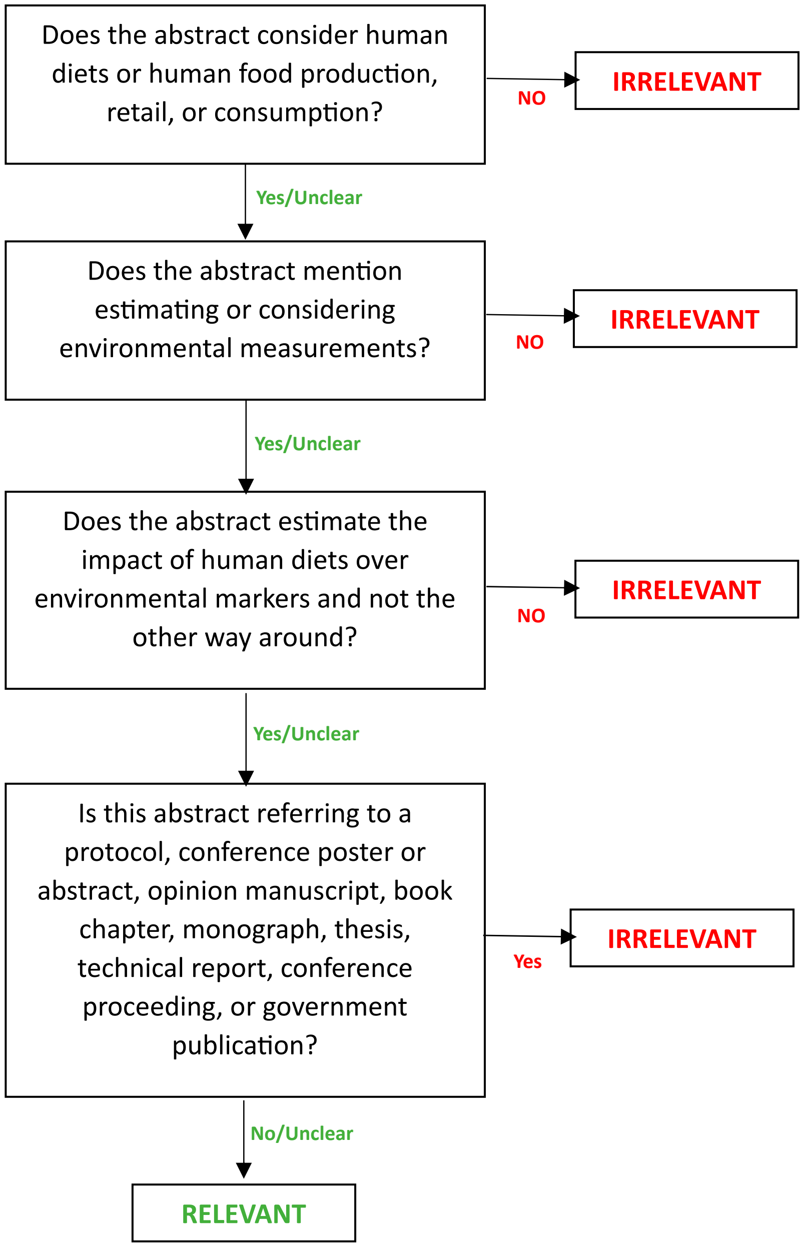

Figure S1. Progress while training the AI tool.

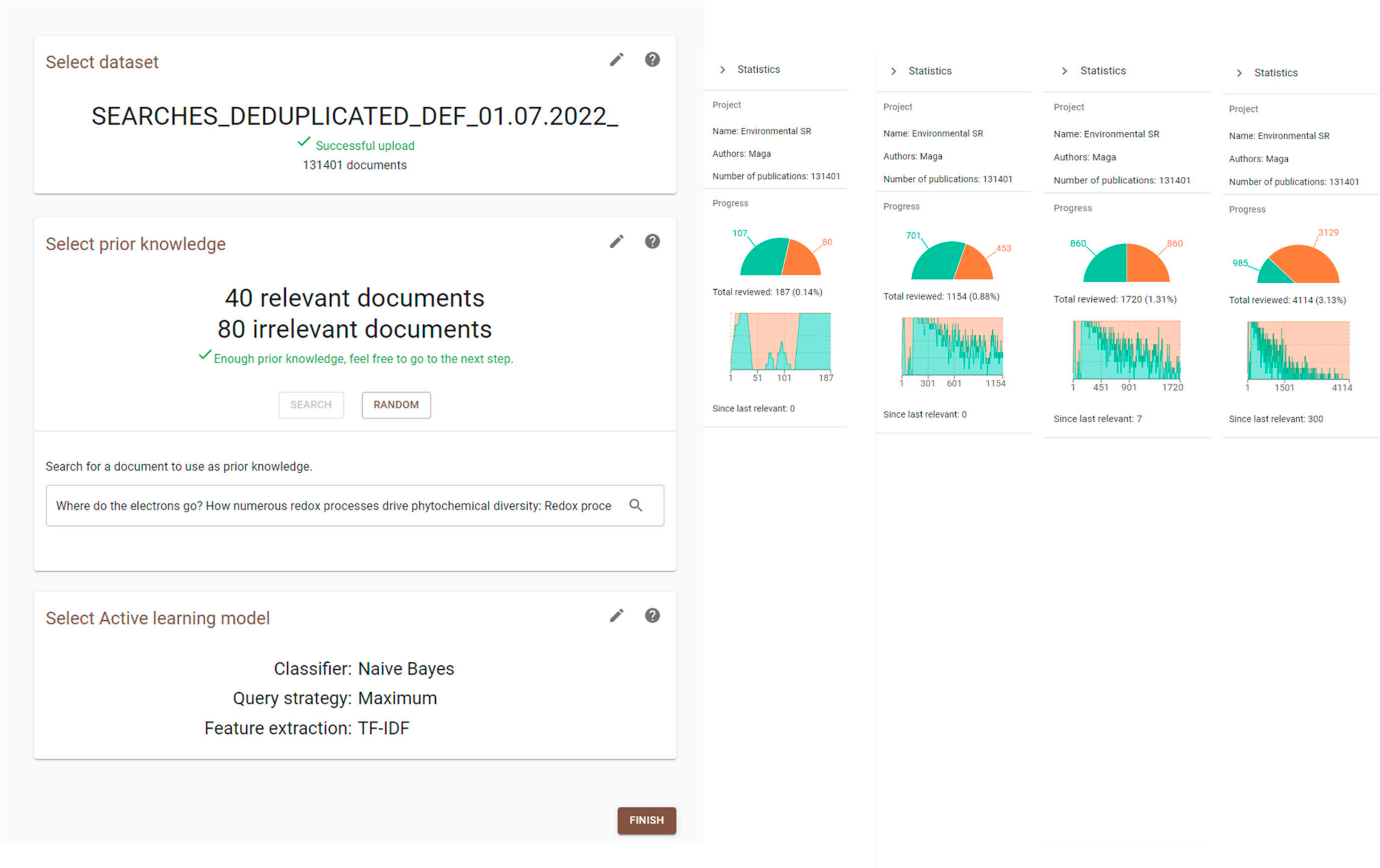

Updated search 27.11.2023

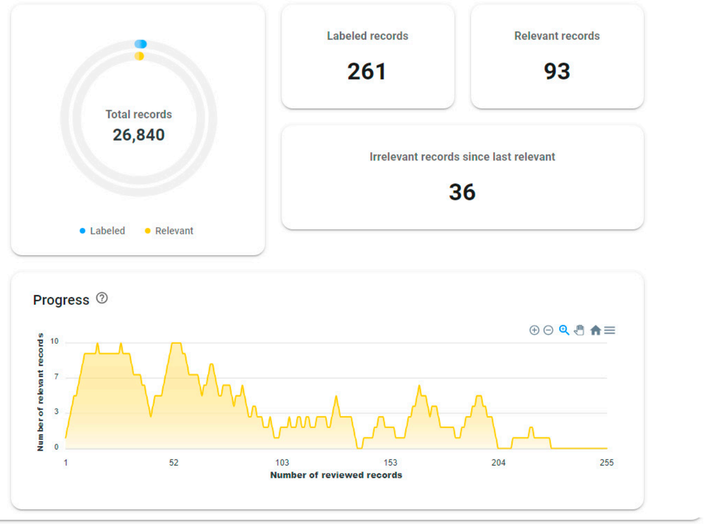

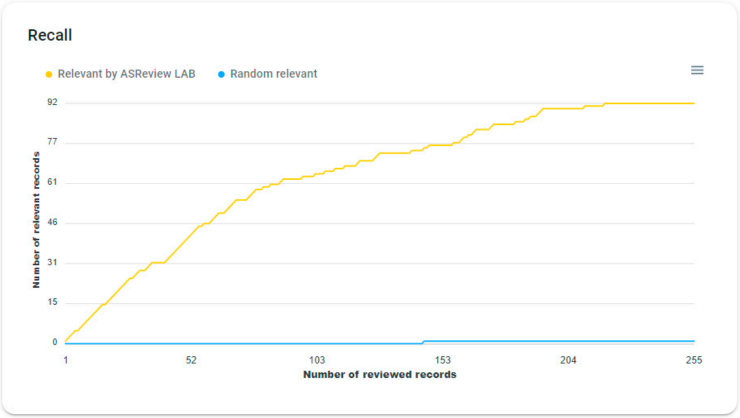

Supplement: Supplementary file 1 [file nutrients-16-03166-s001.zip › Supplementary Material S2 AI training 27.08..2024.pdf]
