# Supplementary material for: What Environmental Metrics Are Used in Scientific Research to Estimate the Impact of Human Diets?
Source: Nutrients. 2024 Sep 19;16(18):3166. doi: 10.3390/nu16183166 (PMC11435316; doi:10.3390/nu16183166)
Supplement: Supplementary file 1 [file nutrients-16-03166-s001.zip › Supplementary Material S3 List of included studies 27.08..2024.pdf]

Supplementary Material S3. List of Included studies

1. Abejón Elías R, Batlle Bayer L, Laso Cortabitarte J, Bala Gala A, Vazquez Rowe I, Larrea Gallegos G, Margallo Blanco M, Cristóbal García J, Puig Vidal R, Fullana i Palmer P, Aldaco García R. Multi-objective optimization of nutritional, environmental and economic aspects of diets applied to the Spanish context. Journal of Health Services Research & Policy 2020 (1)
2. Aceves-Martins M, Bates RL, Craig LC, Chalmers N, Horgan G, Boskamp B, De Roos B. Food-Level Analysis to Identify Dietary Choices With the Highest Nutritional Quality and Lowest Greenhouse Gas Emissions and Price. Frontiers in nutrition. 2022:770.
3. Aceves-Martins M, Bates RL, Craig LC, Chalmers N, Horgan G, Boskamp B, de Roos B. Nutritional Quality, Environmental Impact and Cost of Ultra-Processed Foods: A UK Food-Based Analysis. International journal of environmental research and public health. 2022 Mar 8;19(6):3191.
4. Aceves-Martins M, Bates RL, Craig LC, Chalmers N, Horgan G, Boskamp B, de Roos B. Consumption of foods with the highest nutritional quality, and the lowest GHGE and price, differs between socioeconomic groups in the UK population. Public Health Nutrition. 2023 Oct 31:1-23.
5. Adhikari B, Prapaspongsa T. Environmental sustainability of food consumption in Asia. Sustainability. 2019 Oct 17;11(20):5749.
6. Aguiar DR, da Costa GN, Simões GT, Figueiredo AM. Diet-related greenhouse gas emissions in Brazilian State capital cities. Environmental Science & Policy. 2021 Oct 1;124:542-52.
7. Aleksandrowicz L, Green R, Joy EJ, Harris F, Hillier J, Vetter SH, Smith P, Kulkarni B, Dangour AD, Haines A. Environmental impacts of dietary shifts in India: A modelling study using nationally-representative data. Environment international. 2019 May 1;126:207-15.
8. Alexander P, Rounsevell MD, Dislich C, Dodson JR, Engström K, Moran D. Drivers for global agricultural land use change: The nexus of diet, population, yield and bioenergy. Global Environmental Change. 2015 Nov 1;35:138-47.
9. Ali M, Liu L, Zhang J. Exploring spatio-temporal variations in environmental impacts from eating out in the United Kingdom. Science of The Total Environment. 2021 Dec 20;801:149540.
10. Arrieta EM, Fischer CG, Aguiar S, Geri M, Fernández RJ, Coquet JB, Scavuzzo CM, Rieznik A, León A, González AD, Jobbágy EG. The health, environmental, and economic dimensions of future dietary transitions in Argentina. Sustainability science. 2022 Jan 15:1-7.
11. Arrieta EM, Geri M, Coquet JB, Scavuzzo CM, Zapata ME, González AD. Quality and environmental footprints of diets by socio-economic status in Argentina. Science of the Total Environment. 2021 Dec 20;801:149686.
12. Arrieta EM, Gonzalez AD. Impact of current, National Dietary Guidelines and alternative diets on greenhouse gas emissions in Argentina. Food Policy. 2018 Aug 1;79:58-66.
13. Aston LM, Smith JN, Powles JW. Impact of a reduced red and processed meat dietary pattern on disease risks and greenhouse gas emissions in the UK: a modelling study. BMJ open. 2012 Jan 1;2(5):e001072.
14. Auclair O, Burgos SA. Carbon footprint of Canadian self-selected diets: Comparing intake of foods, nutrients, and diet quality between low-and high-greenhouse gas emission diets. Journal of Cleaner Production. 2021 Sep 20;316:128245.
15. Auma CI, Pradeilles R, Blake MK, Holdsworth M. What can dietary patterns tell us about the nutrition transition and environmental sustainability of diets in Uganda?. Nutrients. 2019 Feb 5;11(2):342.
16. Avetisyan M, Hertel T, Sampson G. Is local food more environmentally friendly? The GHG emissions impacts of consuming imported versus domestically produced food. Environmental and Resource Economics. 2014 Jul;58(3):415-62.

17. Bahn R, EL Labban S, Hwalla N. Impacts of shifting to healthier food consumption patterns on environmental sustainability in MENA countries. Sustainability Science. 2019 Jul;14(4):1131-46.
18. Bajželj B, Richards KS, Allwood JM, Smith P, Dennis JS, Curmi E, Gilligan CA. Importance of food-demand management for climate mitigation. Nature Climate Change. 2014 Oct;4(10):924-9.
19. Ball TS, Vaughan NE, Powell TW, Lovett A, Lenton TM. C-LLAMA 1.0: a traceable model for food, agriculture, and land use. Geoscientific Model Development. 2022 Feb 1;15(2):929-49.
20. Bälter K, Sjörs C, Sjölander A, Gardner C, Hedenus F, Tillander A. Is a diet low in greenhouse gas emissions a nutritious diet?–Analyses of self-selected diets in the LifeGene study. Archives of Public Health. 2017 Dec;75(1):1-9.
21. Barnsley JE, Chandrakumar C, Gonzalez-Fischer C, Eme PE, Bourke BE, Smith NW, Dave LA, McNabb WC, Clark H, Frame DJ, Lynch J. Lifetime climate impacts of diet transitions: a novel climate change accounting perspective. Sustainability. 2021 May 17;13(10):5568.
22. Baroni L, Berati M, Candilera M, Tettamanti M. Total environmental impact of three main dietary patterns in relation to the content of animal and plant food. Foods. 2014 Jul 25;3(3):443-60.
23. Barosh LJ, Lawrence M, Friel S. Towards healthy and sustainable food consumption: an Australian case study.
24. Barre T, Perignon M, Gazan R, Vieux F, Micard V, Amiot MJ, Darmon N. Integrating nutrient bioavailability and co-production links when identifying sustainable diets: how low should we reduce meat consumption?. PLoS One. 2018 Feb 14;13(2):e0191767.
25. Bassi C, Maysels R, Anex R. Declining greenhouse gas emissions in the US diet (2003–2018): Drivers and demographic trends. Journal of Cleaner Production. 2022 Jun 1;351:131465.
26. Batlle-Bayer L, Aldaco R, Bala A, Puig R, Laso J, Margallo M, Vázquez-Rowe I, Antó JM, Fullana-i-Palmer P. Environmental and nutritional impacts of dietary changes in Spain during the COVID-19 lockdown. Science of The Total Environment. 2020 Dec 15;748:141410.
27. Batlle-Bayer L, Bala A, Albertí J, Xifré R, Aldaco R, Fullana-i-Palmer P. Food affordability and nutritional values within the functional unit of a food LCA. An application on regional diets in Spain. Resources, Conservation and Recycling. 2020 Sep 1;160:104856.
28. Batlle-Bayer L, Bala A, Aldaco R, Vidal-Monés B, Colomé R, Fullana-i-Palmer P. An explorative assessment of environmental and nutritional benefits of introducing low-carbon meals to Barcelona schools. Science of The Total Environment. 2021 Feb 20;756:143879.
29. Batlle-Bayer L, Bala A, Lemaire E, Albertí J, García-Herrero I, Aldaco R, Fullana-i-Palmer P. An energy-and nutrient-corrected functional unit to compare LCAs of diets. Science of The Total Environment. 2019 Jun 25;671:175-9.
30. Batlle-Bayer L, Bala A, Roca M, Lemaire E, Aldaco R, Fullana-i-Palmer P. Nutritional and environmental co-benefits of shifting to "Planetary Health" Spanish tapas. Journal of Cleaner Production. 2020 Oct 20;271:122561.
31. Baudry J, Neves F, Lairon D, Allès B, Langevin B, Brunin J, Berthy F, Danquah I, Touvier M, Hercberg S, Amiot MJ. Sustainability analysis of the Mediterranean diet: results from the French NutriNet-Santé study. British Journal of Nutrition. 2023 Jun 26:1-42.
32. Baudry J, Pointereau P, Seconda L, Vidal R, Taupier-Letage B, Langevin B, Allès B, Galan P, Hercberg S, Amiot MJ, Boizot-Szantai C. Improvement of diet sustainability with increased level of organic food in the diet: findings from the BioNutriNet cohort. The American journal of clinical nutrition. 2019 Apr 1;109(4):1173-88.
33. Bayram HM, Ozturkcan SA. Greenhouse gas emissions in the food system: Current and alternative dietary scenarios. Mediterranean Journal of Nutrition and Metabolism. 2022 Jan 1;15(4):463-77.

34. Beckerman JP, Blondin SA, Richardson SA, Rimm EB. Environmental and economic effects of changing to shelf-stable dairy or soy milk for the breakfast in the classroom program. American journal of public health. 2019 May;109(5):736-8.
35. Behrens P, Kiefte-de Jong JC, Bosker T, Rodrigues JF, De Koning A, Tukker A. Evaluating the environmental impacts of dietary recommendations. Proceedings of the National Academy of Sciences. 2017 Dec 19;114(51):13412-7.
36. Belgacem W, Mattas K, Arampatzis G, Baourakis G. Changing dietary behavior for better biodiversity preservation: a preliminary study. Nutrients. 2021 Jun 17;13(6):2076.
37. Benis K, Ferrão P. Potential mitigation of the environmental impacts of food systems through urban and peri-urban agriculture (UPA)–a life cycle assessment approach. Journal of Cleaner Production. 2017 Jan 1;140:784-95.
38. Benvenuti L, De Santis A, Di Sero A, Franco N. Concurrent economic and environmental impacts of food consumption: are low emissions diets affordable?. Journal of Cleaner Production. 2019 Nov 1;236:117645.
39. Benvenuti L, De Santis A, Santesarti F, Tocca L. An optimal plan for food consumption with minimal environmental impact: the case of school lunch menus. Journal of Cleaner Production. 2016 Aug 15;129:704-13.
40. Berardy A, Fresán U, Matos RA, Clarke A, Mejia A, Jaceldo-Siegl K, Sabaté J. Environmental impacts of foods in the adventist health study-2 dietary questionnaire. Sustainability. 2020 Dec 9;12(24):10267.
41. Bertoluci G, Masset G, Gomy C, Mottet J, Darmon N. How to build a standardized country-specific environmental food database for nutritional epidemiology studies. PLoS One. 2016 Apr 7;11(4):e0150617.
42. Beylot A, Secchi M, Cerutti A, Merciai S, Schmidt J, Sala S. Assessing the environmental impacts of EU consumption at macro-scale. Journal of cleaner production. 2019 Apr 10;216:382-93.
43. Bezerra IN, Verde SM, Almeida BD, de Azevedo CV. Carbon Footprint of Away-From-Home Food Consumption in Brazilian Diet. International Journal of Environmental Research and Public Health. 2022 Dec 13;19(24):16708.
44. Biesbroek S, Bueno-de-Mesquita HB, Peeters PH, Verschuren WM, van der Schouw YT, Kramer GF, Tyszler M, Temme EH. Reducing our environmental footprint and improving our health: greenhouse gas emission and land use of usual diet and mortality in EPIC-NL: a prospective cohort study. Environmental Health. 2014 Dec;13(1):1-9.
45. Biesbroek S, Monique Verschuren WM, van der Schouw YT, Sluijs I, Boer J, Temme EH. Identification of data-driven Dutch dietary patterns that benefit the environment and are healthy. Climatic change. 2018 Apr;147(3):571-83.
46. Biesbroek S, Verschuren WM, Boer JM, van de Kamp ME, Van Der Schouw YT, Geelen A, Looman M, Temme EH. Does a better adherence to dietary guidelines reduce mortality risk and environmental impact in the Dutch sub-cohort of the European Prospective Investigation into Cancer and Nutrition?. British Journal of Nutrition. 2017 Jul;118(1):69-80.
47. Biesbroek S, Verschuren WM, Boer JM, van der Schouw YT, Sluijs I, Temme EH. Are our diets getting healthier and more sustainable? Insights from the European Prospective Investigation into Cancer and Nutrition–Netherlands (EPIC-NL) cohort. Public health nutrition. 2019 Nov;22(16):2931-40.
48. Bilali HE, Calabrese G, Iannetta M, Stefanova M, Paoletti F, Ladisa G, Bottalico F, Capone R. Environmental sustainability of typical agro-food products: A scientifically sound and user friendly approach.
49. Blackstone NT, El-Abbadi NH, McCabe MS, Griffin TS, Nelson ME. Linking sustainability to the healthy eating patterns of the Dietary Guidelines for Americans: a modelling study. The Lancet Planetary Health. 2018 Aug 1;2(8):e344-52.

50. Blas A, Garrido A, Willaarts BA. Evaluating the water footprint of the Mediterranean and American diets. Water. 2016 Oct 13;8(10):448.
51. Boehm R, Ver Ploeg M, Wilde PE, Cash SB. Greenhouse gas emissions, total food spending and diet quality by share of household food spending on red meat: results from a nationally representative sample of US households. Public health nutrition. 2019 Jul;22(10):1794-806.
52. Boehm R, Wilde PE, Ver Ploeg M, Costello C, Cash SB. A comprehensive life cycle assessment of greenhouse gas emissions from US household food choices. Food Policy. 2018 Aug 1;79:67-76.
53. Bordoni A. Insight into the Sustainability of the Mediterranean Diet: The Water Footprint of the Recommended Italian Diet. Nutrients. 2023 May 5;15(9):2204.
54. Boyer D, Ramaswami A. Comparing urban food system characteristics and actions in US and Indian cities from a multi-environmental impact perspective: Toward a streamlined approach. Journal of Industrial Ecology. 2020 Aug;24(4):841-54.
55. Boyer D, Ramaswami A. What is the contribution of city-scale actions to the overall food system's environmental impacts?: Assessing water, greenhouse gas, and land impacts of future urban food scenarios. Environmental science & technology. 2017 Oct 17;51(20):12035-45.
56. Boyer D, Sarkar J, Ramaswami A. Diets, food miles, and environmental sustainability of urban food systems: Analysis of nine indian cities. Earth's Future. 2019 Aug;7(8):911-22.
57. Bozeman III JF, Ashton WS, Theis TL. Distinguishing environmental impacts of household food-spending patterns among US demographic groups. Environmental Engineering Science. 2019 Jul 1;36(7):763-77.
58. Bozeman III JF, Springfield S, Theis TL. Meeting EAT-lancet food consumption, nutritional, and environmental health standards: A US case study across racial and ethnic subgroups. Environmental Justice. 2020 Oct 1;13(5):160-72.
59. Bozeman, J.F., Bozeman, R. and Theis, T.L., 2020. Overcoming climate change adaptation barriers: A study on food–energy–water impacts of the average American diet by demographic group. Journal of Industrial Ecology, 24(2), pp.383-399.
60. Bradley P. Environmental impacts of food retail: a framework method and case application. Journal of Cleaner Production. 2016 Feb 1;113:153-66.
61. Broekema R, Tyszler M, van't Veer P, Kok FJ, Martin A, Lluch A, Blonk HT. Future-proof and sustainable healthy diets based on current eating patterns in the Netherlands. The American journal of clinical nutrition. 2020 Nov;112(5):1338-47.
62. Bruins MJ, Létinois U. Adequate vitamin D intake cannot be achieved within carbon emission limits unless food is fortified: A simulation study. Nutrients. 2021 Feb 11;13(2):592.
63. Bruno M, Thomsen M, Pulselli FM, Patrizi N, Marini M, Caro D. The carbon footprint of Danish diets. Climatic Change. 2019 Oct;156(4):489-507.
64. Bryan T, Hicks A, Barrett B, Middlecamp C. An environmental impact calculator for 24-h diet recalls. Sustainability. 2019 Dec 3;11(23):6866.
65. Cai H, Biesbroek S, Wen X, Fan S, van't Veer P, Talsma EF. Environmental footprints of Chinese foods and beverages: Literature-based construction of a LCA database. Data in brief. 2022 Jun 1;42:108244.
66. Camanzi L, Alikadic A, Compagnoni L, Merloni E. The impact of greenhouse gas emissions in the EU food chain: A quantitative and economic assessment using an environmentally extended input-output approach. Journal of Cleaner Production. 2017 Jul 20;157:168-76.
67. Cambeses-Franco C, Feijoo G, Moreira MT, González-García S. Co-benefits of the EAT-Lancet diet for environmental protection in the framework of the Spanish dietary pattern. Science of The Total Environment. 2022 Aug 25;836:155683.
68. Cambeses-Franco C, González-García S, Feijoo G, Moreira MT. Driving commitment to sustainable food policies within the framework of American and European dietary guidelines. Science of the Total Environment. 2022 Feb 10;807:150894.

69. Cambeses-Franco C, González-García S, Feijoo G, Moreira MT. Encompassing health and nutrition with the adherence to the environmentally sustainable New Nordic Diet in Southern Europe. Journal of Cleaner Production. 2021 Dec 10;327:129470.
70. Cambeses-Franco C, González-García S, Feijoo G, Moreira MT. Is the Paleo diet safe for health and the environment?. Science of The Total Environment. 2021 Aug 10;781:146717.
71. Candy S, Turner G, Larsen K, Wingrove K, Steenkamp J, Friel S, Lawrence M. Modelling the food availability and environmental impacts of a shift towards consumption of healthy dietary patterns in Australia. Sustainability. 2019 Dec 12;11(24):7124.
72. Cao Y, Chai L, Yan X, Liang Y. Drivers of the growing water, carbon and ecological footprints of the Chinese diet from 1961 to 2017. International journal of environmental research and public health. 2020 Mar;17(5):1803.
73. Castaldi S, Dembska K, Antonelli M, Petersson T, Piccolo MG, Valentini R. The positive climate impact of the Mediterranean diet and current divergence of Mediterranean countries towards less climate sustainable food consumption patterns. Scientific Reports. 2022 May 25;12(1):1-9.
74. Castañé S, Antón A. Assessment of the nutritional quality and environmental impact of two food diets: A Mediterranean and a vegan diet. Journal of cleaner production. 2017 Nov 20;167:929-37.
75. Cederberg C, Hedenus F, Wirsenius S, Sonesson U. Trends in greenhouse gas emissions from consumption and production of animal food products–implications for long-term climate targets. Animal. 2013 Feb;7(2):330-40.
76. Cederberg C, Persson UM, Schmidt S, Hedenus F, Wood R. Beyond the borders–burdens of Swedish food consumption due to agrochemicals, greenhouse gases and land-use change. Journal of Cleaner Production. 2019 Mar 20;214:644-52.
77. Chapa J, Farkas B, Bailey RL, Huang JY. Evaluation of environmental performance of dietary patterns in the United States considering food nutrition and satiety. Science of The Total Environment. 2020 Jun 20;722:137672.
78. Chaudhary A, Krishna V. Country-specific sustainable diets using optimization algorithm. Environmental science & technology. 2019 May 30;53(13):7694-703.
79. Chen C, Chaudhary A, Mathys A. Dietary change scenarios and implications for environmental, nutrition, human health and economic dimensions of food sustainability. Nutrients. 2019 Apr 16;11(4):856.
80. Churak P, Sranacharoenpong K, Mungcharoen T. Environmental consequences related to nutritional status of Thai populations. Journal of Public Health. 2021 Aug;29:879-84.
81. Clark M, Tilman D. Comparative analysis of environmental impacts of agricultural production systems, agricultural input efficiency, and food choice. Environmental Research Letters. 2017 Jun 16;12(6):064016.
82. Clay N, Charlton K, Stefoska-Needham A, Heffernan E, Hassan HI, Jiang X, Stanford J, Lambert K. What is the climate footprint of therapeutic diets for people with chronic kidney disease? Results from an Australian analysis. Journal of Human Nutrition and Dietetics. 2023 Dec;36(6):2246-55.
83. Clora F, Yu W, Baudry G, Costa L. Impacts of supply-side climate change mitigation practices and trade policy regimes under dietary transition: the case of European agriculture. Environmental Research Letters. 2021 Dec 3;16(12):124048.
84. Cobiac LJ, Scarborough P. Modelling the health co-benefits of sustainable diets in the UK, France, Finland, Italy and Sweden. European journal of clinical nutrition. 2019 Apr;73(4):624-33.
85. Coelho CR, Pernollet F, van der Werf HM. Environmental life cycle assessment of diets with improved omega-3 fatty acid profiles. PLoS One. 2016 Aug 9;11(8):e0160397.

86. Corrado S, Luzzani G, Trevisan M, Lamastra L. Contribution of different life cycle stages to the greenhouse gas emissions associated with three balanced dietary patterns. Science of the Total Environment. 2019 Apr 10;660:622-30.
87. Crenna E, Sinkko T, Sala S. Biodiversity impacts due to food consumption in Europe. Journal of cleaner production. 2019 Aug 1;227:378-91.
88. Curi-Quinto K, Unar-Munguía M, Rodríguez-Ramírez S, Rivera JA, Fanzo J, Willett W, Röös E. Sustainability of Diets in Mexico: Diet Quality, Environmental Footprint, Diet Cost, and Sociodemographic Factors. Frontiers in nutrition. 2022;9.
89. da Silva, J.T., Garzillo, J.M.F., Rauber, F., Kluczkovski, A., Rivera, X.S., da Cruz, G.L., Frankowska, A., Martins, C.A., da Costa Louzada, M.L., Monteiro, C.A. and Reynolds, C., 2021. Greenhouse gas emissions, water footprint, and ecological footprint of food purchases according to their degree of processing in Brazilian metropolitan areas: a time-series study from 1987 to 2018. The Lancet Planetary Health, 5(11), pp.e775-e785.
90. Dahmani J, Nicklaus S, Grenier JM, Marty L. Nutritional quality and greenhouse gas emissions of vegetarian and non-vegetarian primary school meals: A case study in Dijon, France. Frontiers in nutrition. 2022 Oct 10;9:997144.
91. Das K, Gerbens-Leenes PW, Nonhebel S. The water footprint of food and cooking fuel: A case study of self-sufficient rural India. Journal of Cleaner Production. 2021 Jan 25;281:125255.
92. de Carvalho AM, César CL, Fisberg RM, Marchioni DM. Excessive meat consumption in Brazil: diet quality and environmental impacts. Public Health Nutrition. 2013 Oct;16(10):1893-9.
93. De Laurentiis V, Hunt DV, Lee SE, Rogers CD. EATS: a life cycle-based decision support tool for local authorities and school caterers. The International Journal of Life Cycle Assessment. 2019 Jul;24(7):1222-38.
94. de Pee S, Hardinsyah R, Jalal F, Kim BF, Semba RD, Deptford A, Fanzo JC, Ramsing R, Nachman KE, McKenzie S, Bloem MW. Balancing a sustained pursuit of nutrition, health, affordability and climate goals: exploring the case of Indonesia. The American journal of clinical nutrition. 2021 Nov;114(5):1686-97.
95. de Ruiter H, Kastner T, Nonhebel S. European dietary patterns and their associated land use: Variation between and within countries. Food policy. 2014 Feb 1;44:158-66.
96. Dekker E, Zijp MC, van de Kamp ME, Temme EH, van Zelm R. A taste of the new ReCiPe for life cycle assessment: Consequences of the updated impact assessment method on food product LCAs. The International Journal of Life Cycle Assessment. 2020 Dec;25(12):2315-24.
97. Deng G, Xu Y, Yu Z. Accounting and change trend analysis of food production water footprint in China. Water Policy. 2018 Aug 1;20(4):758-76.
98. Detzel A, Krüger M, Busch M, Blanco-Gutiérrez I, Varela C, Manners R, Bez J, Zannini E. Life cycle assessment of animal-based foods and plant-based protein-rich alternatives: an environmental perspective. Journal of the Science of Food and Agriculture. 2021 Jul 7.
99. Dhar AR, Oita A, Matsubae K. The effect of religious dietary cultures on food nitrogen and phosphorus footprints: A case study of India. Nutrients. 2021 Jun;13(6):1926.
100. Di Paola A, Rulli MC, Santini M. Human food vs. animal feed debate. A thorough analysis of environmental footprints. Land use policy. 2017 Sep 1;67:652-9.
101. Dixon KA, Michelsen MK, Carpenter CL. Modern diets and the health of our planet: An investigation into the environmental impacts of food choices. Nutrients. 2023 Jan 30;15(3):692.
102. Djanibekov N, Frohberg K, Djanibekov U. Income-based projections of water footprint of food consumption in Uzbekistan. Global and planetary change. 2013 Nov 1;110:130-42.
103. Dogbe W, Revoredo-Giha C. Nutritional and environmental assessment of increasing the content of fruit and vegetables in the UK diet. Sustainability. 2021 Jan 21;13(3):1076.
104. Donati M, Menozzi D, Zighetti C, Rosi A, Zinetti A, Scazzina F. Towards a sustainable diet combining economic, environmental and nutritional objectives. Appetite. 2016 Nov 1;106:48-57.

105. Dong L, Zhang G, Li X, Liu H, Pei H, Song X, Liao Q, Liu Y, Niu Y. Dominant drivers of the increasing environmental footprint of changing diets in China. Journal of Cleaner Production. 2021 Aug 20;312:127694.
106. Dong Y, Miller SA. Assessing the lifecycle greenhouse gas (GHG) emissions of perishable food products delivered by the cold chain in China. Journal of Cleaner Production. 2021 Jun 20;303:126982.
107. Doran-Browne NA, Eckard RJ, Behrendt R, Kingwell RS. Nutrient density as a metric for comparing greenhouse gas emissions from food production. Climatic Change. 2015 Mar;129(1):73-87.
108. Drew J, Cleghorn C, Macmillan A, Mizdrak A. Healthy and climate-friendly eating patterns in the New Zealand context. Environmental health perspectives. 2020 Jan 22;128(1):017007.
109. Drewnowski A, Rehm CD, Martin A, Verger EO, Voinnesson M, Imbert P. Energy and nutrient density of foods in relation to their carbon footprint. The American journal of clinical nutrition. 2015 Jan 1;101(1):184-91.
110. Drewnowski A. Measures and metrics of sustainable diets with a focus on milk, yogurt, and dairy products. Nutrition reviews. 2018 Jan 1;76(1):21-8.
111. Duro JA, Lauk C, Kastner T, Erb KH, Haberl H. Global inequalities in food consumption, cropland demand and land-use efficiency: A decomposition analysis. Global Environmental Change. 2020 Sep 1;64:102124.
112. Eberle U, Fels J. Environmental impacts of German food consumption and food losses. The International Journal of Life Cycle Assessment. 2016 May;21(5):759-72.
113. Eini-Zinab H, Shoaibinobarian N, Ranjbar G, Ostad AN, Sobhani SR. Association between the socio-economic status of households and a more sustainable diet. Public Health Nutrition. 2021 Dec;24(18):6566-74.
114. Eini-Zinab H, Sobhani SR, Rezazadeh A. Designing a healthy, low-cost and environmentally sustainable food basket: an optimisation study. Public Health Nutrition. 2021 May;24(7):1952-61.
115. Eshel G, Shepon A, Makov T, Milo R. Land, irrigation water, greenhouse gas, and reactive nitrogen burdens of meat, eggs, and dairy production in the United States. Proceedings of the National Academy of Sciences. 2014 Aug 19;111(33):11996-2001.
116. Esteve-Llorens X, Darriba C, Moreira MT, Feijoo G, González-García S. Towards an environmentally sustainable and healthy Atlantic dietary pattern: Life cycle carbon footprint and nutritional quality. Science of the Total Environment. 2019 Jan 1;646:704-15.
117. Esteve-Llorens X, Moreira MT, Feijoo G, González-García S. Linking environmental sustainability and nutritional quality of the Atlantic diet recommendations and real consumption habits in Galicia (NW Spain). Science of The Total Environment. 2019 Sep 15;683:71-9.
118. Eustachio Colombo P, Elinder LS, Lindroos AK, Parlesak A. Designing Nutritionally Adequate and Climate-Friendly Diets for Omnivorous, Pescatarian, Vegetarian and Vegan Adolescents in Sweden Using Linear Optimization. Nutrients. 2021 Jul 22;13(8):2507.
119. Eustachio Colombo P, Milner J, Scheelbeek PF, Taylor A, Parlesak A, Kastner T, Nicholas O, Elinder LS, Dangour AD, Green R. Pathways to "5-a-day": Modeling the health impacts and environmental footprints of meeting the target for fruit and vegetable intake in the United Kingdom. The American journal of clinical nutrition. 2021 Aug;114(2):530-9.
120. Eustachio Colombo P, Patterson E, Lindroos AK, Parlesak A, Elinder LS. Sustainable and acceptable school meals through optimization analysis: an intervention study. Nutrition journal. 2020 Dec;19(1):1-5.
121. Eustachio Colombo P, Patterson E, Schäfer Elinder L, Lindroos AK, Sonesson U, Darmon N, Parlesak A. Optimizing school food supply: integrating environmental, health, economic, and cultural dimensions of diet sustainability with linear programming. International journal of environmental research and public health. 2019 Sep;16(17):3019.

122. Fanelli RM. The interactions between the structure of the food supply and the impact of livestock production on the environment. A multivariate analysis for understanding the differences and the analogies across European Union countries. Calitatea. 2018 Dec 1;19(167):131-9.
123. Farchi S, De Sario M, Lapucci E, Davoli M, Michelozzi P. Meat consumption reduction in Italian regions: health co-benefits and decreases in GHG emissions. PloS one. 2017 Aug 15;12(8):e0182960.
124. Ferrari M, Benvenuti L, Rossi L, De Santis A, Sette S, Martone D, Piccinelli R, Le Donne C, Leclercq C, Turrini A. Could dietary goals and climate change mitigation be achieved through optimized diet? The experience of modeling the national food consumption data in Italy. Frontiers in nutrition. 2020 May 4;7:48.
125. Filippin D, Sarni AR, Rizzo G, Baroni L. Environmental Impact of Two Plant-Based, Isocaloric and Isoproteic Diets: The Vegan Diet vs. the Mediterranean Diet. International Journal of Environmental Research and Public Health. 2023 Feb 21;20(5):3797.
126. Foong A, Pradhan P, Frör O, Kropp JP. Adjusting agricultural emissions for trade matters for climate change mitigation. Nature Communications. 2022 Jun 9;13(1):1-0.
127. Frankowska A, Jeswani HK, Azapagic A. Environmental impacts of vegetables consumption in the UK. Science of The Total Environment. 2019 Sep 10;682:80-105.
128. Frankowska A, Jeswani HK, Azapagic A. Life cycle environmental impacts of fruits consumption in the UK. Journal of environmental management. 2019 Oct 15;248:109111.
129. Frehner A, Cardinaals RP, de Boer IJ, Muller A, Schader C, van Selm B, Van Hal O, Pestoni G, Rohrmann S, Herrero M, van Zanten HH. The compatibility of circularity and national dietary recommendations for animal products in five European countries: a modelling analysis on nutritional feasibility, climate impact, and land use. The Lancet Planetary Health. 2022 Jun 1;6(6):e475-83.
130. Frehner A, Van Zanten HH, Schader C, De Boer IJ, Pestoni G, Rohrmann S, Muller A. How food choices link sociodemographic and lifestyle factors with sustainability impacts. Journal of Cleaner Production. 2021 Jun 1;300:126896.
131. Fresán U, Craig WJ, Martínez-González MA, Bes-Rastrollo M. Nutritional quality and health effects of low environmental impact diets: The "seguimiento universidad de navarra"(sun) Cohort. Nutrients. 2020 Aug 9;12(8):2385.
132. Fresán U, Martínez-González MA, Sabaté J, Bes-Rastrollo M. Global sustainability (health, environment and monetary costs) of three dietary patterns: Results from a Spanish cohort (the SUN project). BMJ open. 2019 Feb 1;9(2):e021541.
133. Fresán U, Martínez-Gonzalez MA, Sabaté J, Bes-Rastrollo M. The Mediterranean diet, an environmentally friendly option: Evidence from the Seguimiento Universidad de Navarra (SUN) cohort. Public health nutrition. 2018 Jun;21(8):1573-82.
134. Fresán U, Martínez-González MA, Segovia-Siapco G, Sabaté J, Bes-Rastrollo M. A three-dimensional dietary index (nutritional quality, environment and price) and reduced mortality: The "Seguimiento Universidad de Navarra" cohort. Preventive Medicine. 2020 Aug 1;137:106124.
135. Fresán U, Mejia MA, Craig WJ, Jaceldo-Siegl K, Sabaté J. Meat analogs from different protein sources: a comparison of their sustainability and nutritional content. Sustainability. 2019 Jun 12;11(12):3231.
136. Galeana-Pizaña JM, Couturier S, Monsivais-Huertero A. Assessing food security and environmental protection in Mexico with a GIS-based Food Environmental Efficiency index. Land use policy. 2018 Jul 1;76:442-54.
137. Galli A, Iha K, Halle M, El Bilali H, Grunewald N, Eaton D, Capone R, Debs P, Bottalico F. Mediterranean countries' food consumption and sourcing patterns: an ecological footprint viewpoint. Science of the Total Environment. 2017 Feb 1;578:383-91.

138. Gallo I, Landro N, La Grassa R, Turconi A. Food Recommendations for Reducing Water Footprint. Sustainability. 2022 Mar 24;14(7):3833.
139. García S, Bouzas C, Mateos D, Pastor R, Álvarez L, Rubín M, Martínez-González MÁ, Salas-Salvadó J, Corella D, Goday A, Martínez JA. Carbon dioxide (CO2) emissions and adherence to Mediterranean diet in an adult population: the Mediterranean diet index as a pollution level index. Environmental Health. 2023 Jan 5;22(1):1.
140. García S, Pastor R, Monserrat-Mesquida M, Álvarez-Álvarez L, Rubín-García M, Martínez-González MÁ, Salas-Salvadó J, Corella D, Fitó M, Martínez JA, Tojal-Sierra L. Ultra-processed foods consumption as a promoting factor of greenhouse gas emissions, water, energy, and land use: A longitudinal assessment. Science of the Total Environment. 2023 Sep 15;891:164417.
141. García S, Pastor R, Monserrat-Mesquida M, Álvarez-Álvarez L, Rubín-García M, Martínez-González MÁ, Salas-Salvadó J, Corella D, Goday A, Martínez JA, Alonso-Gómez ÁM. Metabolic syndrome criteria and severity and carbon dioxide (CO2) emissions in an adult population. Globalization and health. 2023 Jul 13;19(1):50.
142. García-Herrero L, Costello C, De Menna F, Schreiber L, Vittuari M. Eating away at sustainability. Food consumption and waste patterns in a US school canteen. Journal of Cleaner Production. 2021 Jan 10;279:123571.
143. Garzillo JM, Machado PP, Leite FH, Steele EM, Poli VF, Louzada ML, Levy RB, Monteiro CA. Carbon footprint of the Brazilian diet. Revista de saude publica. 2021 Dec 8;55.
144. Garzillo JM, Poli VF, Leite FH, Steele EM, Machado PP, Louzada ML, Levy RB, Monteiro CA. Ultra-processed food intake and diet carbon and water footprints: a national study in Brazil. Revista de Saúde Pública. 2022 Feb 28;56:6.
145. Gazan R, Maillot M, Reboul E, Darmon N. Pulses twice a week in replacement of meat modestly increases diet sustainability. Nutrients. 2021 Aug 31;13(9):3059.
146. Gephart JA, Davis KF, Emery KA, Leach AM, Galloway JN, Pace ML. The environmental cost of subsistence: optimizing diets to minimize footprints. Science of the Total Environment. 2016 May 15;553:120-7.
147. Germani A, Vitiello V, Giusti AM, Pinto A, Donini LM, del Balzo V. Environmental and economic sustainability of the Mediterranean Diet. International journal of food sciences and nutrition. 2014 Dec 1;65(8):1008-12.
148. Geyik Ö, Hadjikakou M, Bryan BA. Climate-friendly and nutrition-sensitive interventions can close the global dietary nutrient gap while reducing GHG emissions. Nature Food. 2023 Jan;4(1):61-73.
149. Goldstein B, Moses R, Sammons N, Birkved M. Potential to curb the environmental burdens of American beef consumption using a novel plant-based beef substitute. PloS one. 2017 Dec 6;12(12):e0189029.
150. González CA, Bonet C, de Pablo M, Sanchez MJ, Salamanca-Fernandez E, Dorronsoro M, Amiano P, Huerta JM, Chirlaque MD, Ardanaz E, Barricarte A. Greenhouse gases emissions from the diet and risk of death and chronic diseases in the EPIC-Spain cohort. European Journal of Public Health. 2021 Feb;31(1):130-5.
151. González-García S, Esteve-Llorens X, González-García R, González L, Feijoo G, Moreira MT, Leis R. Environmental assessment of menus for toddlers serviced at nursery canteen following the Atlantic diet recommendations. Science of The Total Environment. 2021 May 20;770:145342.
152. González-García S, González-García R, Vázquez LG, Moreira MT, Leis R. Tracking the environmental footprints of institutional restaurant service in nursery schools. Science of The Total Environment. 2020 Aug 1;728:138939.
153. González-García S, Green RF, Scheelbeek PF, Harris F, Dangour AD. Dietary recommendations in Spain–affordability and environmental sustainability?. Journal of cleaner production. 2020 May 1;254:120125.

154. Gormaz T, Cortés S, Tiboni-Oschilewski O, Weisstaub G. The Chilean Diet: Is It Sustainable?. Nutrients. 2022 Jul 28;14(15):3103.
155. Grasso AC, Besselink JJ, Tyszler M, Bruins MJ. The Potential of Food Fortification as an Enabler of More Environmentally Sustainable, Nutritionally Adequate Diets. Nutrients. 2023 May 25;15(11):2473.
156. Grasso AC, Olthof MR, van Dooren C, Broekema R, Visser M, Brouwer IA. Protein for a healthy future: how to increase protein intake in an environmentally sustainable way in older adults in the Netherlands. The Journal of nutrition. 2021 Jan;151(1):109-19.
157. Grasso AC, Olthof MR, van Dooren C, Roca M, Gili M, Visser M, Cabout M, Bot M, Penninx BW, van Grootheest G, Kohls E. Effect of food-related behavioral activation therapy on food intake and the environmental impact of the diet: results from the MooDFOOD prevention trial. European journal of nutrition. 2020 Sep;59(6):2579-91.
158. Green R, Milner J, Dangour AD, Haines A, Chalabi Z, Markandya A, Spadaro J, Wilkinson P. The potential to reduce greenhouse gas emissions in the UK through healthy and realistic dietary change. Climatic Change. 2015 Mar;129(1):253-65.
159. Green RF, Joy EJ, Harris F, Agrawal S, Aleksandrowicz L, Hillier J, Macdiarmid JI, Milner J, Vetter SH, Smith P, Haines A. Greenhouse gas emissions and water footprints of typical dietary patterns in India. Science of the total environment. 2018 Dec 1;643:1411-8.
160. Grosso G, Fresán U, Bes-Rastrollo M, Marventano S, Galvano F. Environmental impact of dietary choices: role of the Mediterranean and other dietary patterns in an Italian cohort. International journal of environmental research and public health. 2020 Mar;17(5):1468.
161. Grummon AH, Lee CJ, Robinson TN, Rimm EB, Rose D. Simple dietary substitutions can reduce carbon footprints and improve dietary quality across diverse segments of the US population. Nature Food. 2023 Oct 26:1-2.
162. Gualtieri P, Marchetti M, Frank G, Cianci R, Bigioni G, Colica C, Soldati L, Moia A, De Lorenzo A, Di Renzo L. Exploring the Sustainable Benefits of Adherence to the Mediterranean Diet during the COVID-19 Pandemic in Italy. Nutrients. 2022 Dec 26;15(1):110.
163. Hadjikakou M. Trimming the excess: environmental impacts of discretionary food consumption in Australia. Ecological Economics. 2017 Jan 1;131:119-28.
164. Hallström E, Bajzelj B, Håkansson N, Sjons J, Åkesson A, Wolk A, Sonesson U. Dietary climate impact: Contribution of foods and dietary patterns by gender and age in a Swedish population. Journal of Cleaner Production. 2021 Jul 15;306:127189.
165. Hallström E, Gee Q, Scarborough P, Cleveland DA. A healthier US diet could reduce greenhouse gas emissions from both the food and health care systems. Climatic Change. 2017 May;142(1):199-212.
166. Hallström E, Röös E, Börjesson P. Sustainable meat consumption: A quantitative analysis of nutritional intake, greenhouse gas emissions and land use from a Swedish perspective. Food Policy. 2014 Aug 1;47:81-90.
167. Han A, Chai L, Liu P. How much environmental burden does the shifting to nutritional diet bring? Evidence of dietary transformation in rural China. Environmental Science & Policy. 2023 Jul 1;145:129-38.
168. Hansen AD, Kuramochi T, Wicke B. The status of corporate greenhouse gas emissions reporting in the food sector: An evaluation of food and beverage manufacturers. Journal of Cleaner Production. 2022 May 18:132279.
169. Hanssen OJ, Vold M, Schakenda V, Tufte PA, Møller H, Olsen NV, Skaret J. Environmental profile, packaging intensity and food waste generation for three types of dinner meals. Journal of Cleaner Production. 2017 Jan 20;142:395-402.
170. Harris F, Green RF, Joy EJ, Kayatz B, Haines A, Dangour AD. The water use of Indian diets and socio-demographic factors related to dietary blue water footprint. Science of the Total Environment. 2017 Jun 1;587:128-36.

171. Hatjiathanassiadou M, Souza SR, Nogueira JP, Oliveira LD, Strasburg VJ, Rolim PM, Seabra LM. Environmental impacts of university restaurant menus: A case study in Brazil. Sustainability. 2019 Sep 20;11(19):5157.
172. Haupt M, Sanchís JR, Polo GC, García-Segovia P, Pellicer NS. Aproximación a la integración de la huella de carbono y aspectos nutricionales para un consumo sostenible de alimentos. Rev Esp Nutr Comunitaria. 2016 Mar 9;22(1):2-9.
173. Hawkins J, Ma C, Schilizzi S, Zhang F. China's changing diet and its impacts on greenhouse gas emissions: an index decomposition analysis. Australian Journal of Agricultural and Resource Economics. 2018 Jan;62(1):45-64.
174. He G, Geng C, Zhao Y, Wang J, Jiang S, Zhu Y, Wang Q, Wang L, Mu X. Food habit and climate change impacts on agricultural water security during the peak population period in China. Agricultural Water Management. 2021 Dec 1;258:107211.
175. He P, Baiocchi G, Feng K, Hubacek K, Yu Y. Environmental impacts of dietary quality improvement in China. Journal of environmental management. 2019 Jun 15;240:518-26.
176. He P, Cai B, Baiocchi G, Liu Z. Drivers of GHG emissions from dietary transition patterns in China: Supply versus demand options. Journal of Industrial Ecology. 2021 Jun;25(3):707-19.
177. Heard BR, Bandekar M, Vassar B, Miller SA. Comparison of life cycle environmental impacts from meal kits and grocery store meals. Resources, Conservation and Recycling. 2019 Aug 1;147:189-200.
178. Heerschop SN, Biesbroek S, Temme EH, Ocké MC. Can healthy and sustainable dietary patterns that fit within current dutch food habits be identified?. Nutrients. 2021 Apr 2;13(4):1176.
179. Heerschop SN, Kanellopoulos A, Biesbroek S, van ‘t Veer P. Shifting towards optimized healthy and sustainable Dutch diets: Impact on protein quality. European Journal of Nutrition. 2023 Mar 23:1-4.
180. Heller MC, Walchale A, Heard BR, Hoey L, Khoury CK, De Haan S, Burra DD, Duong TT, Osiemo J, Trinh TH, Jones AD. Environmental analyses to inform transitions to sustainable diets in developing countries: case studies for Vietnam and Kenya. The International Journal of Life Cycle Assessment. 2020 Jul;25(7):1183-96.
181. Heller MC, Willits-Smith A, Meyer R, Keoleian GA, Rose D. Greenhouse gas emissions and energy use associated with production of individual self-selected US diets. Environmental Research Letters. 2018 Mar 20;13(4):044004.
182. Hendrie GA, Baird D, Ridoutt B, Hadjikakou M, Noakes M. Overconsumption of energy and excessive discretionary food intake inflates dietary greenhouse gas emissions in Australia. Nutrients. 2016 Oct 31;8(11):690.
183. Hendrie GA, Ridoutt BG, Wiedmann TO, Noakes M. Greenhouse gas emissions and the Australian diet—comparing dietary recommendations with average intakes. Nutrients. 2014 Jan 8;6(1):289-303.
184. Hitaj C, Rehkamp S, Canning P, Peters CJ. Greenhouse gas emissions in the United States food system: current and healthy diet scenarios. Environmental Science & Technology. 2019 Apr 23;53(9):5493-503.
185. Hjorth T, Huseinovic E, Hallström E, Strid A, Johansson I, Lindahl B, Sonesson U, Winkvist A. Changes in dietary carbon footprint over ten years relative to individual characteristics and food intake in the Västerbotten Intervention Programme. Scientific reports. 2020 Jan 8;10(1):1-4.
186. Hobbs DA, Durrant C, Elliott J, Givens DI, Lovegrove JA. Diets containing the highest levels of dairy products are associated with greater eutrophication potential but higher nutrient intakes and lower financial cost in the United Kingdom. European journal of nutrition. 2020 Apr;59(3):895-908.

187. Hoolohan C, Berners-Lee M, McKinstry-West J, Hewitt CN. Mitigating the greenhouse gas emissions embodied in food through realistic consumer choices. Energy Policy. 2013 Dec 1;63:1065-74.
188. Hor R, Ly P, Putra AS, Ishizaki R, Ahamed T, Noguchi R. Estimation of Carbon Dioxide Emissions from a Traditional Nutrient-Rich Cambodian Diet Food Production System Using Life Cycle Assessment. Sustainability. 2021 Mar 25;13(7):3660.
189. Horgan GW, Perrin A, Whybrow S, Macdiarmid JI. Achieving dietary recommendations and reducing greenhouse gas emissions: modelling diets to minimise the change from current intakes. International Journal of Behavioral Nutrition and Physical Activity. 2016 Dec;13(1):1-1.
190. Hu Y, Su M, Sun M, Wang Y, Xu X, Wang L, Zhang L. Environmental footprints of improving dietary quality of Chinese rural residents: A modeling study. Resources, Conservation and Recycling. 2022 Apr 1;179:106074.
191. Huang G, Yao G, Zhao J, Lisk MD, Yu C, Zhang X. The environmental and socioeconomic trade-offs of importing crops to meet domestic food demand in China. Environmental Research Letters. 2019 Sep 20;14(9):094021.
192. Huseinovic E, Ohlin M, Winkvist A, Bertz F, Sonesson U, Brekke HK. Does diet intervention in line with nutrition recommendations affect dietary carbon footprint? Results from a weight loss trial among lactating women. European journal of clinical nutrition. 2017 Oct;71(10):1241-5.
193. Hwalla N, Jomaa L, Hachem F, Kharroubi S, Hamadeh R, Nasreddine L, Naja F. Promoting Sustainable and Healthy Diets to Mitigate Food Insecurity Amidst Economic and Health Crises in Lebanon. Frontiers in nutrition. 2021:351.
194. Hyland JJ, Henchion M, McCarthy M, McCarthy SN. The climatic impact of food consumption in a representative sample of Irish adults and implications for food and nutrition policy. Public Health Nutrition. 2017 Mar;20(4):726-38.
195. Jalava M, Guillaume JH, Kummu M, Porkka M, Siebert S, Varis O. Diet change and food loss reduction: What is their combined impact on global water use and scarcity?. Earth's Future. 2016 Mar;4(3):62-78.
196. Jalava M, Kummu M, Porkka M, Siebert S, Varis O. Diet change—a solution to reduce water use?. Environmental research letters. 2014 Jul 31;9(7):074016.
197. Jennings R, Henderson AD, Phelps A, Janda KM, van den Berg AE. Five US Dietary Patterns and Their Relationship to Land Use, Water Use, and Greenhouse Gas Emissions: Implications for Future Food Security. Nutrients. 2023 Jan 1;15(1):215.
198. Karaçil Ermumcu MŞ, Çıtar Dazıroğlu ME, Erdoğan Gövez N, Acar Tek N. Evaluation of personal water footprint components in Turkey: factors associated with obesity and food consumption. International Journal of Environmental Health Research. 2023 Jan 25:1-1.
199. Karlsson JO, Röös E. Resource-efficient use of land and animals—Environmental impacts of food systems based on organic cropping and avoided food-feed competition. Land Use Policy. 2019 Jun 1;85:63-72.
200. Kassem R, Jepsen MR, Salhofer SP. The water consumption of different diets in Denmark. Journal of Cleaner Production. 2021 Mar 1;286:124938.
201. Kastner T, Rivas MJ, Koch W, Nonhebel S. Global changes in diets and the consequences for land requirements for food. Proceedings of the National Academy of Sciences. 2012 May 1;109(18):6868-72.
202. Kemaloglu M, Öner N, Soylu M. Environmental impacts and diet quality of popular diet models compared to Turkey's national nutrition guidelines. Nutrition & Dietetics. 2023 Apr;80(2):183-91.
203. Kesse-Guyot E, Allès B, Brunin J, Fouillet H, Dussiot A, Berthy F, Perraud E, Hercberg S, Julia C, Mariotti F, Deschasaux-Tanguy M. Environmental impacts associated with UPF

consumption: which food chain stages matter the most? Findings from a representative sample of French adults. medRxiv. 2022 May 31:2022-05.
204. Kesse-Guyot E, Allès B, Brunin J, Langevin B, Fouillet H, Dussiot A, Berthy F, Reuzé A, Perraud E, Rebouillat P, Touvier M. Environmental pressures and pesticide exposure associated with an increase in the share of plant-based foods in the diet. Scientific Reports. 2023 Nov 7;13(1):19317.
205. Kesse-Guyot E, Pointereau P, Brunin J, Perraud E, Toujgani H, Berthy F, Allès B, Touvier M, Lairon D, Mariotti F, Baudry J. Trade-offs between water use and greenhouse gas emissions related to food systems: an optimization study in French adults. medRxiv. 2023:2023-03.
206. Kesse-Guyot E, Rebouillat P, Brunin J, Langevin B, Allès B, Touvier M, Hercberg S, Fouillet H, Huneau JF, Mariotti F, Lairon D. Environmental and nutritional analysis of the EAT-Lancet diet at the individual level: insights from the NutriNet-Santé study. Journal of Cleaner Production. 2021 May 10;296:126555.
207. Kesse-Guyot, E., Fouillet, H., Baudry, J., Dussiot, A., Langevin, B., Allès, B., Rebouillat, P., Brunin, J., Touvier, M., Hercberg, S. and Lairon, D., 2021. Halving food-related greenhouse gas emissions can be achieved by redistributing meat consumption: progressive optimization Results of the NutriNet-Santé cohort. Science of the Total Environment, 789, p.147901.
208. Kidd B, Mackay S, Vandevijvere S, Swinburn B. Cost and greenhouse gas emissions of current, healthy, flexitarian and vegan diets in Aotearoa (New Zealand). BMJ Nutrition, Prevention & Health. 2021;4(1):275.
209. Kim D, Parajuli R, Thoma GJ. Life cycle assessment of dietary patterns in the United States: a full food supply chain perspective. Sustainability. 2020 Feb 20;12(4):1586.
210. Kirwan LB, Walton J, Flynn A, Nugent AP, Kearney J, Holden NM, McNulty BA. Assessment of the Environmental Impact of Food Consumption in Ireland—Informing a Transition to Sustainable Diets. Nutrients. 2023 Feb 16;15(4):981.
211. Kluczkovski A, Menezes CA, da Silva JT, Bastos L, Lait R, Cook J, Cruz B, Cerqueira B, Lago RM, Gomes AN, Ladeia AM. An environmental and nutritional evaluation of school food menus in Bahia, Brazil that contribute to local public policy to promote sustainability. Nutrients. 2022 Apr 6;14(7):1519.
212. Koelman L, Huybrechts I, Biesbroek S, van 't Veer P, Schulze MB, Aleksandrova K. Dietary Choices Impact on Greenhouse Gas Emissions: Determinants and Correlates in a Sample of Adults from Eastern Germany. Sustainability. 2022 Mar 24;14(7):3854.
213. Kovacs B, Miller L, Heller MC, Rose D. The carbon footprint of dietary guidelines around the world: a seven country modeling study. Nutrition journal. 2021 Dec;20(1):1-0.
214. Kramer GF, Martinez EV, Espinoza-Orias ND, Cooper KA, Tyszler M, Blonk H. Comparing the performance of bread and breakfast cereals, dairy, and meat in nutritionally balanced and sustainable diets. Front. Nutr.. 2018 Jun 7;5:51.
215. Kramer GF, Tyszler M, van't Veer P, Blonk H. Decreasing the overall environmental impact of the Dutch diet: how to find healthy and sustainable diets with limited changes. Public health nutrition. 2017 Jun;20(9):1699-709.
216. Kucukvar M, Onat NC, Abdella GM, Tatari O. Assessing regional and global environmental footprints and value added of the largest food producers in the world. Resources, Conservation and Recycling. 2019 May 1;144:187-97.
217. Lachat C, Raneri JE, Smith KW, Kolsteren P, Van Damme P, Verzelen K, Penafiel D, Vanhove W, Kennedy G, Hunter D, Odhiambo FO. Dietary species richness as a measure of food biodiversity and nutritional quality of diets. Proceedings of the National Academy of Sciences. 2018 Jan 2;115(1):127-32.
218. Lacour C, Seconda L, Allès B, Hercberg S, Langevin B, Pointereau P, Lairon D, Baudry J, Kesse-Guyot E. Environmental impacts of plant-based diets: how does organic food consumption contribute to environmental sustainability?. Frontiers in nutrition. 2018:8.

219. Laine JE, Huybrechts I, Gunter MJ, Ferrari P, Weiderpass E, Tsilidis K, Aune D, Schulze MB, Bergmann M, Temme EH, Boer JM. Co-benefits from sustainable dietary shifts for population and environmental health: an assessment from a large European cohort study. The Lancet Planetary Health. 2021 Nov 1;5(11):e786-96.
220. Lares-Michel M, Housni FE, Aguilera Cervantes VG, Carrillo P, Michel Nava RM, Llanes Cañedo C. Eat well to fight obesity… and save water: the water footprint of different diets and caloric intake and its relationship with adiposity. Frontiers in nutrition. 2021:382.
221. Lares-Michel M, Housni FE, Aguilera Cervantes VG, Reyes-Castillo Z, Michel Nava RM, Llanes Cañedo C, López Larios MD. The water footprint and nutritional implications of diet change in Mexico: a principal component analysis. European Journal of Nutrition. 2022 Apr 19:1-26.
222. Lares-Michel M, Housni FE, Aguilera Cervantes VG. A quantitative estimation of the water footprint of the Mexican diet, corrected for washing and cooking water. Food Security. 2021 Aug;13(4):849-74.
223. Laroche PC, Schulp CJ, Kastner T, Verburg PH. Telecoupled environmental impacts of current and alternative Western diets. Global Environmental Change. 2020 May 1;62:102066.
224. Larrea-Gallegos G, Vázquez-Rowe I. Optimization of the environmental performance of food diets in Peru combining linear programming and life cycle methods. Science of the Total Environment. 2020 Jan 10;699:134231.
225. Lassen AD, Nordman M, Christensen LM, Beck AM, Trolle E. Guidance for healthy and more climate-friendly diets in nursing homes—scenario analysis based on a municipality's food procurement. Nutrients. 2021 Dec 17;13(12):4525.
226. Lauk C, Kaufmann L, Theurl MC, Wittmann F, Eder M, Hörtenhuber S, Freyer B, Krausmann F. Demand side options to reduce greenhouse gas emissions and the land footprint of urban food systems: A scenario analysis for the City of Vienna. Journal of Cleaner Production. 2022 Jul 20;359:132064.
227. Li H, Wu T, Wang X, Qi Y. The greenhouse gas footprint of China's food system: an analysis of recent trends and future scenarios. Journal of Industrial Ecology. 2016 Aug;20(4):803-17.
228. Li Y, Zhong H, Shan Y, Hang Y, Wang D, Zhou Y, Hubacek K. Changes in global food consumption increase GHG emissions despite efficiency gains along global supply chains. Nature Food. 2023 Jun 15:1-3.
229. Liang Y, Han A, Chai L, Zhi H. Using the machine learning method to study the environmental footprints embodied in chinese diet. International Journal of Environmental Research and Public Health. 2020 Oct;17(19):7349.
230. Lin B, Xie X. CO2 emissions of China's food industry: an input–output approach. Journal of Cleaner Production. 2016 Jan 20;112:1410-21.
231. Lindroos AK, Hallström E, Moraeus L, Strid A, Winkvist A. Dietary Greenhouse Gas Emissions and Diet Quality in a Cross-Sectional Study of Swedish Adolescents. The American Journal of Clinical Nutrition. 2023 Nov 1;118(5):956-65.
232. Liu C, Zou C, Wang Q, Hayashi Y, Yasunari T. Impact assessment of human diet changes with rapid urbanization on regional nitrogen and phosphorus flows—a case study of the megacity Shanghai. Environmental Science and Pollution Research. 2014 Feb;21(3):1905-14.
233. Liu J, Ma K, Ciais P, Polasky S. Reducing human nitrogen use for food production. Scientific reports. 2016 Jul 22;6(1):1-4.
234. Liu Y, Lin J, Li H, Huang R, Han H. Driving forces of food consumption water footprint in North China. Water. 2021 Mar 16;13(6):810.
235. Liyanapathirana NN, Grech A, Li M, Malik A, Lenzen M, Raubenheimer D. Nutrient-sensitive approach for sustainability assessment of different dietary patterns in Australia. The American Journal of Clinical Nutrition. 2022 Apr;115(4):1048-58.

236. Liyanapathirana NN, Grech A, Li M, Malik A, Lenzen M, Raubenheimer D. Nutrient-sensitive approach for sustainability assessment of Australian macronutrient dietary recommendations. The American Journal of Clinical Nutrition. 2023 Feb 1;117(2):298-307.
237. Liyanapathirana NN, Grech A, Li M, Malik A, Ribeiro R, Burykin T, Lenzen M, Raubenheimer D. Nutritional, environmental and economic impacts of ultra-processed food consumption in Australia. Public Health Nutrition. 2023 Oct 26:1-1.
238. Long Y, Hu R, Yin T, Wang P, Liu J, Muhammad T, Chen X, Li Y. Spatial-temporal footprints assessment and driving mechanism of China household diet based on CHNS. Foods. 2021 Aug 11;10(8):1858.
239. López-Olmedo N, Stern D, Bakhtsiyarava M, Pérez-Ferrer C, Langellier B. Greenhouse Gas Emissions Associated With the Mexican Diet: Identifying Social Groups With the Largest Carbon Footprint. Frontiers in nutrition. 2022:559.
240. Lucas E, Galán-Martín Á, Pozo C, Guo M, Guillén-Gosálbez G. Global environmental and nutritional assessment of national food supply patterns: Insights from a data envelopment analysis approach. Science of the Total Environment. 2021 Feb 10;755:142826.
241. Lukas M, Rohn H, Lettenmeier M, Liedtke C, Wiesen K. The nutritional footprint–integrated methodology using environmental and health indicators to indicate potential for absolute reduction of natural resource use in the field of food and nutrition. Journal of cleaner production. 2016 Sep 20;132:161-70.
242. Mackie C, Wemhoff AP. Comparing greenhouse gas emissions associated with food away from home versus food at home in the United States. Journal of Cleaner Production. 2020 Jul 1;260:120930.
243. Majewski E, Komerska A, Kwiatkowski J, Malak-Rawlikowska A, Wąs A, Sulewski P, Gołaś M, Pogodzińska K, Lecoeur JL, Tocco B, Török Á. Are short food supply chains more environmentally sustainable than long chains? A life cycle assessment (LCA) of the eco-efficiency of food chains in selected EU countries. Energies. 2020 Sep 16;13(18):4853.
244. Martin M, Brandão M. Evaluating the environmental consequences of Swedish food consumption and dietary choices. Sustainability. 2017 Dec 1;9(12):2227.
245. Martinez S, del Mar Delgado M, Marin RM, Alvarez S. How do dietary choices affect the environment? The nitrogen footprint of the European Union and other dietary options. Environmental Science & Policy. 2019 Nov 1;101:204-10.
246. Martinez S, Delgado MD, Marin RM, Alvarez S. Carbon footprint of school lunch menus adhering to the Spanish dietary guidelines. Carbon management. 2020 Jul 3;11(4):427-39.
247. Marty L, de Lauzon-Guillain B, Nicklaus S. Short-and Mid-Term Impacts of COVID-19 Outbreak on the Nutritional Quality and Environmental Impact of Diet. Frontiers in nutrition. 2022:314.
248. Masino T, Colombo PE, Reis K, Tetens I, Parlesak A. Climate-friendly, health-promoting, and culturally acceptable diets for German adult omnivores, pescatarians, vegetarians, and vegans–a linear programming approach. Nutrition. 2023 May 1;109:111977.
249. Masset G, Soler LG, Vieux F, Darmon N. Identifying sustainable foods: the relationship between environmental impact, nutritional quality, and prices of foods representative of the French diet. Journal of the Academy of Nutrition and Dietetics. 2014 Jun 1;114(6):862-9.
250. Masset G, Vieux F, Verger EO, Soler LG, Touazi D, Darmon N. Reducing energy intake and energy density for a sustainable diet: a study based on self-selected diets in French adults. The American journal of clinical nutrition. 2014 Jun 1;99(6):1460-9.
251. Mehlig K, Blomqvist I, Klingberg S, Bianchi M, Sjons J, Hunsberger M, Lissner L. Secular trends in diet-related greenhouse gas emission estimates since 2000–a shift towards sustainable diets in Sweden. Public health nutrition. 2021 Aug;24(12):3916-21.
252. Meier T, Christen O, Semler E, Jahreis G, Voget-Kleschin L, Schrode A, Artmann M. Balancing virtual land imports by a shift in the diet. Using a land balance approach to assess

the sustainability of food consumption. Germany as an example. Appetite. 2014 Mar 1;74:20-34.
253. Meier T, Christen O. Environmental impacts of dietary recommendations and dietary styles: Germany as an example. Environmental science & technology. 2013 Jan 15;47(2):877-88.
254. Meier T, Christen O. Gender as a factor in an environmental assessment of the consumption of animal and plant-based foods in Germany. The International Journal of Life Cycle Assessment. 2012 Jun;17(5):550-64.
255. Mekonnen MM, Fulton J. The effect of diet changes and food loss reduction in reducing the water footprint of an average American. Water international. 2018 Aug 18;43(6):860-70.
256. Mertens E, Kuijsten A, Geleijnse JM, Boshuizen HC, Feskens EJ, van't Veer P. FFQ versus repeated 24-h recalls for estimating diet-related environmental impact. Nutrition journal. 2019 Dec;18(1):1-2.
257. Mertens E, Kuijsten A, Kanellopoulos A, Dofková M, Mistura L, D'addezio L, Turrini A, Dubuisson C, Havard S, Trolle E, Eckl M. Improving health and carbon footprints of European diets using a benchmarking approach. Public health nutrition. 2021 Feb;24(3):565-75.
258. Mertens E, Kuijsten A, van Zanten HH, Kaptijn G, Dofková M, Mistura L, D'Addezio L, Turrini A, Dubuisson C, Havard S, Trolle E. Dietary choices and environmental impact in four European countries. Journal of Cleaner Production. 2019 Nov 10;237:117827.
259. Mesquita C, Carvalho M. The carbon footprint of common vegetarian and non-vegetarian meals in Portugal: an estimate, comparison, and analysis. The International Journal of Life Cycle Assessment. 2023 Apr 4:1-5.
260. Michalský M, Hooda PS. Greenhouse gas emissions of imported and locally produced fruit and vegetable commodities: A quantitative assessment. Environmental Science & Policy. 2015 Apr 1;48:32-43.
261. Milner J, Green R, Dangour AD, Haines A, Chalabi Z, Spadaro J, Markandya A, Wilkinson P. Health effects of adopting low greenhouse gas emission diets in the UK. BMJ open. 2015 Apr 1;5(4):e007364.
262. Milner J, Joy EJ, Green R, Harris F, Aleksandrowicz L, Agrawal S, Smith P, Haines A, Dangour AD. Projected health effects of realistic dietary changes to address freshwater constraints in India: a modelling study. The lancet Planetary health. 2017 Apr 1;1(1):e26-32.
263. Moberg E, Karlsson Potter H, Wood A, Hansson PA, Röös E. Benchmarking the Swedish diet relative to global and national environmental targets—Identification of indicator limitations and data gaps. Sustainability. 2020 Feb 14;12(4):1407.
264. Mogensen L, Heusale H, Sinkko T, Poutanen K, Sözer N, Hermansen JE, Knudsen MT. Potential to reduce GHG emissions and land use by substituting animal-based proteins by foods containing oat protein concentrate. Journal of Cleaner Production. 2020 Nov 20;274:122914.
265. Mohareb EA, Heller MC, Guthrie PM. Cities' role in mitigating United States food system greenhouse gas emissions. Environmental science & technology. 2018 May 2;52(10):5545-54.
266. Monetti S, Pregernig M, Speck M, Langen N, Bienge K. Assessing the impact of individual nutrition on biodiversity: A conceptual framework for the selection of indicators targeted at the out-of-home catering sector. Ecological Indicators. 2021 Jul 1;126:107620.
267. Monsivais P, Scarborough P, Lloyd T, Mizdrak A, Luben R, Mulligan AA, Wareham NJ, Woodcock J. Greater accordance with the Dietary Approaches to Stop Hypertension dietary pattern is associated with lower diet-related greenhouse gas production but higher dietary costs in the United Kingdom. The American journal of clinical nutrition. 2015 Jul 1;102(1):138-45.
268. Mrówczyńska-Kamińska A, Bajan B, Pawłowski KP, Genstwa N, Zmyślona J. Greenhouse gas emissions intensity of food production systems and its determinants. PLoS One. 2021 Apr 30;16(4):e0250995.
269. Muller A, Schader C, El-Hage Scialabba N, Brüggemann J, Isensee A, Erb KH, Smith P, Klocke P, Leiber F, Stolze M, Niggli U. Strategies for feeding the world more sustainably with organic agriculture. Nature communications. 2017 Nov 14;8(1):1-3.

270. Murakami K, Livingstone MB. Greenhouse gas emissions of self-selected diets in the UK and their association with diet quality: is energy under-reporting a problem?. Nutrition journal. 2018 Dec;17(1):1-0.
271. Musicus AA, Wang DD, Janiszewski M, Eshel G, Blondin SA, Willett W, Stampfer MJ. Health and environmental impacts of plant-rich dietary patterns: a US prospective cohort study. The Lancet Planetary Health. 2022 Nov 1;6(11):e892-900.
272. Naja F, Hwalla N, El Zouhbi A, Abbas N, Chamieh MC, Nasreddine L, Jomaa L. Changes in environmental footprints associated with dietary intake of Lebanese adolescents between the years 1997 and 2009. Sustainability. 2020 Jun 2;12(11):4519.
273. Naja F, Ismail LC, Abbas N, Saleh S, Ali HI. Adherence to the Mediterranean diet and its association with environmental footprints among women of childbearing age in the United Arab Emirates. European Journal of Nutrition. 2022 Mar 1:1-5.
274. Naja F, Ismail LC, Abbas N, Saleh S, Ali HI. Adherence to the Mediterranean diet and its association with environmental footprints among women of childbearing age in the United Arab Emirates. European Journal of Nutrition. 2022 Aug;61(5):2585-99.
275. Naja F, Itani L, Hamade R, Chamieh MC, Hwalla N. Mediterranean diet and its environmental footprints amid nutrition transition: the case of Lebanon. Sustainability. 2019 Nov 26;11(23):6690.
276. Naja F, Jomaa L, Itani L, Zidek J, El Labban S, Sibai AM, Hwalla N. Environmental footprints of food consumption and dietary patterns among Lebanese adults: a cross-sectional study. Nutrition journal. 2018 Dec;17(1):1-1.
277. Nakamura S, Iida A, Nakatani J, Shimizu T, Ono Y, Watanabe S, Noda K, Kitalong C. Global land use of diets in a small island community: a case study of Palau in the Pacific. Environmental Research Letters. 2021 Jun 17;16(6):065016.
278. Neira DP, Fernández XS, Rodríguez DC, Montiel MS, Cabeza MD. Analysis of the transport of imported food in Spain and its contribution to global warming. Renewable Agriculture and Food Systems. 2016 Feb;31(1):37-48.
279. Nguyen SD, Biesbroek S, Le TD, Feskens EJ, Brouwer ID, Talsma EF. Environmental impact and nutrient adequacy of derived dietary patterns in Vietnam. Frontiers in Nutrition. 2023 Jul 6;10:986241.
280. Nordborg M, Davis J, Cederberg C, Woodhouse A. Freshwater ecotoxicity impacts from pesticide use in animal and vegetable foods produced in Sweden. Science of the Total Environment. 2017 Mar 1;581:448-59.
281. Notarnicola B, Tassielli G, Renzulli PA, Castellani V, Sala S. Environmental impacts of food consumption in Europe. Journal of cleaner production. 2017 Jan 1;140:753-65.
282. O'Malley K, Willits-Smith A, Rose D. Popular diets as selected by adults in the United States show wide variation in carbon footprints and diet quality. The American Journal of Clinical Nutrition. 2023 Apr 1;117(4):701-8.
283. Oita A, Nagano I, Matsuda H. Food nitrogen footprint reductions related to a balanced Japanese diet. Ambio. 2018 Apr;47(3):318-26.
284. Oita A, Wirasenjaya F, Liu J, Webeck E, Matsubae K. Trends in the food nitrogen and phosphorus footprints for Asia's giants: China, India, and Japan. Resources, Conservation and Recycling. 2020 Jun 1;157:104752.
285. Osei-Owusu AK, Towa E, Thomsen M. Exploring the pathways towards the mitigation of the environmental impacts of food consumption. Science of The Total Environment. 2022 Feb 1;806:150528.
286. Pairotti MB, Cerutti AK, Martini F, Vesce E, Padovan D, Beltramo R. Energy consumption and GHG emission of the Mediterranean diet: a systemic assessment using a hybrid LCA-IO method. Journal of Cleaner Production. 2015 Sep 15;103:507-16.

287. Pang J, Li X, Li X, Chen X, Wang H. Research on the relationship between prices of agricultural production factors, food consumption prices, and agricultural carbon emissions: Evidence from china's provincial panel data. Energies. 2021 Jan;14(11):3136.
288. Pang Z, Yan D, Wang T, Kong Y. Disparities and drivers of the water footprint of food consumption in China. Environmental Science and Pollution Research. 2021 Nov;28(44):62461-73.
289. Paris JM, Falkenberg T, Nöthlings U, Heinzel C, Borgemeister C, Escobar N. Changing dietary patterns is necessary to improve the sustainability of Western diets from a One Health perspective. Science of the Total Environment. 2022 Mar 10;811:151437.
290. Park GW, Kim JY, Lee MH, Im Yun J, Park KH. Comparing greenhouse gas emissions and nutritional values based on Korean suggested meal plans and modified vegan meal plans. Journal of Animal Science and Technology. 2020 Jan;62(1):64.
291. Park YS, Egilmez G, Kucukvar M. Emergy and end-point impact assessment of agricultural and food production in the United States: A supply chain-linked Ecologically-based Life Cycle Assessment. Ecological indicators. 2016 Mar 1;62:117-37.
292. Perignon M, Vieux F, Verger EO, Bricas N, Darmon N. Dietary environmental impacts of French adults are poorly related to their income levels or food insecurity status. European Journal of Nutrition. 2023 May 16:1-3.
293. Perignon, M., Masset, G. and Ferrari, G., Tangui Barré, Florent Vieux, Matthieu Maillot, Marie-Josèphe Amiot, and Nicole Darmon. 2016."How Low Can Dietary Greenhouse Gas Emissions Be Reduced without Impairing Nutritional Adequacy, Affordability and Acceptability of the Diet? A Modelling Study to Guide Sustainable Food Choices.". Public Health Nutrition, 19(14), pp.2662-74.
294. Perraud E, Wang J, Salomé M, Mariotti F, Kesse-Guyot E. Dietary protein consumption profiles show contrasting impacts on environmental and health indicators. Science of the Total Environment. 2023 Jan 15;856:159052.
295. Philipp S, Vercalsteren A, José AF, Mathieu S, Boonen K, Maarten C, Giovanni M, Zoboli R, Cathy M. Driving Forces of Changing Environmental Pressures from Consumption in the European Food System.
296. Pitt S, Sjöblom L, Bälter K, Trolle Lagerros Y, Bonn SE. The effect of an app-based dietary intervention on diet-related greenhouse gas emissions–results from a randomized controlled trial. International Journal of Behavioral Nutrition and Physical Activity. 2023 Oct 11;20(1):123.
297. Poinsot R, Vieux F, Maillot M, Darmon N. Number of meal components, nutritional guidelines, vegetarian meals, avoiding ruminant meat: what is the best trade-off for improving school meal sustainability?. European Journal of Nutrition. 2022 Mar 24:1-6.
298. Pollock BD, Willits-Smith AM, Heller MC, Bazzano LA, Rose D. Do diets with higher carbon footprints increase the risk of mortality? A population-based simulation study using self-selected diets from the USA. Public Health Nutrition. 2022 Mar 31:1-7.
299. Pradhan P, Reusser DE, Kropp JP. Embodied greenhouse gas emissions in diets. PloS one. 2013 May 15;8(5):e62228.
300. Prag AA, Henriksen CB. Transition from animal-based to plant-based food production to reduce greenhouse gas emissions from agriculture—the case of Denmark. Sustainability. 2020 Oct 6;12(19):8228.
301. Rancilio G, Gibin D, Blaco A, Casagrandi R. Low-GHG culturally acceptable diets to reduce individual carbon footprint by 20%. Journal of Cleaner Production. 2022 Mar 1;338:130623.
302. Read QD, Hondula KL, Muth MK. Biodiversity effects of food system sustainability actions from farm to fork. Proceedings of the National Academy of Sciences. 2022 Apr 12;119(15):e2113884119.

303. Reguant-Closa A, Roesch A, Lansche J, Nemecek T, Lohman TG, Meyer NL. The environmental impact of the athlete's plate nutrition education tool. Nutrients. 2020 Aug 18;12(8):2484.
304. Reynolds CJ, Horgan GW, Whybrow S, Macdiarmid JI. Healthy and sustainable diets that meet greenhouse gas emission reduction targets and are affordable for different income groups in the UK. Public health nutrition. 2019 Jun;22(8):1503-17.
305. Reynolds CJ, Piantadosi J, Buckley JD, Weinstein P, Boland J. Evaluation of the environmental impact of weekly food consumption in different socio-economic households in Australia using environmentally extended input–output analysis. Ecological Economics. 2015 Mar 1;111:58-64.
306. Ribal J, Fenollosa ML, García-Segovia P, Clemente G, Escobar N, Sanjuán N. Designing healthy, climate friendly and affordable school lunches. The International Journal of Life Cycle Assessment. 2016 May;21(5):631-45.
307. Ridoutt B, Anastasiou K, Baird D, Garcia JN, Hendrie G. Cropland footprints of Australian dietary choices. Nutrients. 2020 Apr 25;12(5):1212.
308. Ridoutt B, Baird D, Hendrie GA. Diets with Higher Vegetable Intake and Lower Environmental Impact: Evidence from a Large Australian Population Health Survey. Nutrients. 2022 Apr 5;14(7):1517.
309. Ridoutt B, Baird D, Hendrie GA. Diets within environmental limits: The climate impact of current and recommended Australian diets. Nutrients. 2021 Mar 29;13(4):1122.
310. Ridoutt B, Baird D, Navarro J, Hendrie GA. Pesticide toxicity footprints of Australian dietary choices. Nutrients. 2021 Nov 29;13(12):4314.
311. Ridoutt BG, Baird D, Anastasiou K, Hendrie GA. An assessment of the water use associated with Australian diets using a planetary boundary framework. Public Health Nutrition. 2021 Apr;24(6):1570-5.
312. Ridoutt BG, Baird D, Anastasiou K, Hendrie GA. Diet quality and water scarcity: evidence from a large Australian population health survey. Nutrients. 2019 Aug 9;11(8):1846.
313. Ridoutt BG, Baird D, Hendrie GA. The role of dairy foods in lower greenhouse gas emission and higher diet quality dietary patterns. European Journal of Nutrition. 2021 Feb;60(1):275-85.
314. Rippin HL, Cade JE, Berrang-Ford L, Benton TG, Hancock N, Greenwood DC. Variations in greenhouse gas emissions of individual diets: Associations between the greenhouse gas emissions and nutrient intake in the United Kingdom. Plos one. 2021 Nov 23;16(11):e0259418.
315. Ritchie H, Reay D, Higgins P. Sustainable food security in India—Domestic production and macronutrient availability. PloS one. 2018 Mar 23;13(3):e0193766.
316. Ritchie H, Reay DS, Higgins P. The impact of global dietary guidelines on climate change. Global environmental change. 2018 Mar 1;49:46-55.
317. Rivera XC, Orias NE, Azapagic A. Life cycle environmental impacts of convenience food: Comparison of ready and home-made meals. Journal of cleaner production. 2014 Jun 15;73:294-309.
318. Rizvi S, Pagnutti C, Fraser E, Bauch CT, Anand M. Global land use implications of dietary trends. PloS one. 2018 Aug 8;13(8):e0200781.
319. Röös E, Karlsson H, Witthöft C, Sundberg C. Evaluating the sustainability of diets–combining environmental and nutritional aspects. Environmental Science & Policy. 2015 Mar 1;47:157-66.
320. Rose D, Heller MC, Willits-Smith AM, Meyer RJ. Carbon footprint of self-selected US diets: nutritional, demographic, and behavioral correlates. The American journal of clinical nutrition. 2019 Mar 1;109(3):526-34.
321. Rose D, Willits-Smith AM, Heller MC. Single-item substitutions can substantially reduce the carbon and water scarcity footprints of US diets. The American journal of clinical nutrition. 2022 Feb;115(2):378-87.

322. Rosi A, Biasini B, Donati M, Ricci C, Scazzina F. Adherence to the mediterranean diet and environmental impact of the diet on primary school children living in parma (Italy). International journal of environmental research and public health. 2020 Sep;17(17):6105.
323. Rosi A, Biasini B, Monica E, Rapetti V, Deon V, Scazzina F. Nutritional composition and environmental impact of meals selected in workplace canteens before and after an intervention promoting the adherence to the Mediterranean diet. Nutrients. 2022 Oct 23;14(21):4456.
324. Rosi A, Mena P, Pellegrini N, Turroni S, Neviani E, Ferrocino I, Di Cagno R, Ruini L, Ciati R, Angelino D, Maddock J. Environmental impact of omnivorous, ovo-lacto-vegetarian, and vegan diet. Scientific reports. 2017 Jul 21;7(1):1-9.
325. Rossi L, Ferrari M, Martone D, Benvenuti L, De Santis A. The promotions of sustainable lunch meals in school feeding programs: the case of Italy. Nutrients. 2021 May;13(5):1571.
326. Ruini LF, Ciati R, Pratesi CA, Marino M, Principato L, Vannuzzi E. Working toward healthy and sustainable diets: The "Double Pyramid Model" developed by the Barilla Center for Food and Nutrition to raise awareness about the environmental and nutritional impact of foods. Frontiers in nutrition. 2015 May 4;2:9.
327. Sáez-Almendros S, Obrador B, Bach-Faig A, Serra-Majem L. Environmental footprints of Mediterranean versus Western dietary patterns: beyond the health benefits of the Mediterranean diet. Environmental Health. 2013 Dec;12(1):1-8.
328. Saner D, Beretta C, Jäggi B, Juraske R, Stoessel F, Hellweg S. FoodPrints of households. The International Journal of Life Cycle Assessment. 2016 May;21(5):654-63.
329. Sanjuán N, Stoessel F, Hellweg S. Closing data gaps for LCA of food products: estimating the energy demand of food processing. Environmental science & technology. 2014 Jan 21;48(2):1132-40.
330. Sarkodie SA, Strezov V, Weldekidan H, Asamoah EF, Owusu PA, Doyi IN. Environmental sustainability assessment using dynamic autoregressive-distributed lag simulations—nexus between greenhouse gas emissions, biomass energy, food and economic growth. Science of the total environment. 2019 Jun 10;668:318-32.
331. Saxe H, Jensen JD, Bølling Laugesen SM, Bredie WL. Environmental impact of meal service catering for dependent senior citizens in Danish municipalities. The international journal of life cycle assessment. 2019 Apr;24(4):654-66.
332. Saxe H, Larsen TM, Mogensen L. The global warming potential of two healthy Nordic diets compared with the average Danish diet. Climatic Change. 2013 Jan;116(2):249-62.
333. Saxe H. The New Nordic Diet is an effective tool in environmental protection: it reduces the associated socioeconomic cost of diets. The American journal of clinical nutrition. 2014 May 1;99(5):1117-25.
334. Scarborough P, Allender S, Clarke D, Wickramasinghe K, Rayner M. Modelling the health impact of environmentally sustainable dietary scenarios in the UK. European journal of clinical nutrition. 2012 Jun;66(6):710-5.
335. Scarborough P, Clark M, Cobiac L, Papier K, Knuppel A, Lynch J, Harrington R, Key T, Springmann M. Vegans, vegetarians, fish-eaters and meat-eaters in the UK show discrepant environmental impacts. Nature Food. 2023 Jul;4(7):565-74.
336. Schaubroeck T, Ceuppens S, Luong AD, Benetto E, De Meester S, Lachat C, Uyttendaele M. A pragmatic framework to score and inform about the environmental sustainability and nutritional profile of canteen meals, a case study on a university canteen. Journal of cleaner production. 2018 Jun 20;187:672-86.
337. Scheelbeek P, Green R, Papier K, Knuppel A, Alae-Carew C, Balkwill A, Key TJ, Beral V, Dangour AD. Health impacts and environmental footprints of diets that meet the Eatwell Guide recommendations: analyses of multiple UK studies. BMJ open. 2020 Aug 1;10(8):e037554.
338. Scherer L, Pfister S. Global biodiversity loss by freshwater consumption and eutrophication from Swiss food consumption. Environmental science & technology. 2016 Jul

339. Schmidt X, Azapagic A. Life cycle environmental impacts of ready-made meals considering different cuisines and recipes.
340. Scovronick N, Wilkinson P. The impact of biofuel-induced food-price inflation on dietary energy demand and dietary greenhouse gas emissions. Global environmental change. 2013 Dec 1;23(6):1587-93.
341. Seconda L, Baudry J, Allès B, Boizot-Szantai C, Soler LG, Galan P, Hercberg S, Langevin B, Lairon D, Pointereau P, Kesse-Guyot E. Comparing nutritional, economic, and environmental performances of diets according to their levels of greenhouse gas emissions. Climatic change. 2018 May;148(1):155-72.
342. Seves SM, Verkaik-Kloosterman J, Biesbroek S, Temme EH. Are more environmentally sustainable diets with less meat and dairy nutritionally adequate?. Public Health Nutrition. 2017 Aug;20(11):2050-62.
343. Sinfort C, Perignon M, Drogué S, Amiot MJ. Dataset on potential environmental impacts of water deprivation and land use for food consumption in France and Tunisia. Data in brief. 2019 Dec 1;27:104661.
344. Sjörs C, Hedenus F, Sjölander A, Tillander A, Bälter K. Adherence to dietary recommendations for Swedish adults across categories of greenhouse gas emissions from food. Public health nutrition. 2017 Dec;20(18):3381-93.
345. Sjörs C, Raposo SE, Sjölander A, Bälter O, Hedenus F, Bälter K. Diet-related greenhouse gas emissions assessed by a food frequency questionnaire and validated using 7-day weighed food records. Environmental health. 2016 Dec;15(1):1-9.
346. Sobhani SR, Rezazadeh A, Omidvar N, Eini-Zinab H. Healthy diet: a step toward a sustainable diet by reducing water footprint. Journal of the Science of Food and Agriculture. 2019 Jun;99(8):3769-75.
347. Sokolow, Jessica;Kennedy, Gina;Attwood, Simon. Managing Crop tradeoffs: A methodology for comparing the water footprint and nutrient density of crops for food system sustainability 2019
348. Song G, Gao X, Fullana-i-Palmer P, Lv D, Zhu Z, Wang Y, Bayer LB. Shift from feeding to sustainably nourishing urban China: a crossing-disciplinary methodology for global environment-food-health nexus. Science of The Total Environment. 2019 Jan 10;647:716-24.
349. Song G, Li M, Fullana-i-Palmer P, Williamson D, Wang Y. Dietary changes to mitigate climate change and benefit public health in China. Science of the Total Environment. 2017 Jan 15;577:289-98.
350. Song G, Li M, Semakula HM, Zhang S. Food consumption and waste and the embedded carbon, water and ecological footprints of households in China. Science of the Total Environment. 2015 Oct 1;529:191-7.
351. Song L, Cai H, Zhu T. Large-Scale Microanalysis of US Household Food Carbon Footprints and Reduction Potentials. Environmental Science & Technology. 2021 Nov 3;55(22):15323-32.
352. Soret S, Mejia A, Batech M, Jaceldo-Siegl K, Harwatt H, Sabaté J. Climate change mitigation and health effects of varied dietary patterns in real-life settings throughout North America. The American journal of clinical nutrition. 2014 Jul 1;100(suppl_1):490S-5S.
353. Souissi A, Mtimet N, McCann L, Chebil A, Thabet C. Determinants of Food Consumption Water Footprint in the MENA Region: The Case of Tunisia. Sustainability. 2022 Jan 28;14(3):1539.
354. Souissi A, Mtimet N, Thabet C, Stambouli T, Chebil A. Impact of food consumption on water footprint and food security in Tunisia. Food Security. 2019 Oct;11(5):989-1008.
355. Springmann M, Clark M, Mason-D'Croz D, Wiebe K, Bodirsky BL, Lassaletta L, De Vries W, Vermeulen SJ, Herrero M, Carlson KM, Jonell M. Options for keeping the food system within environmental limits. Nature. 2018 Oct;562(7728):519-25.

356. Springmann M, Clark MA, Rayner M, Scarborough P, Webb P. The global and regional costs of healthy and sustainable dietary patterns: a modelling study. The Lancet Planetary Health. 2021 Nov 1;5(11):e797-807.
357. Springmann M, Godfray HC, Rayner M, Scarborough P. Analysis and valuation of the health and climate change cobenefits of dietary change. Proceedings of the National Academy of Sciences. 2016 Apr 12;113(15):4146-51.
358. Springmann M, Mason-D'Croz D, Robinson S, Garnett T, Godfray HC, Gollin D, Rayner M, Ballon P, Scarborough P. Global and regional health effects of future food production under climate change: a modelling study. The Lancet. 2016 May 7;387(10031):1937-46.
359. Springmann M, Wiebe K, Mason-D'Croz D, Sulser TB, Rayner M, Scarborough P. Health and nutritional aspects of sustainable diet strategies and their association with environmental impacts: a global modelling analysis with country-level detail. The Lancet Planetary Health. 2018 Oct 1;2(10):e451-61.
360. Sranacharoenpong K. The environmental impacts of 12 country-specific food-based dietary guidelines. Journal of Public Health. 2020 Dec;28(6):719-27.
361. Stewart C, Piernas C, Cook B, Jebb SA. Trends in UK meat consumption: Analysis of data from years 1–11 (2008–09 to 2018–19) of the National Diet and Nutrition Survey rolling programme. The Lancet Planetary Health. 2021 Oct 1;5(10):e699-708.
362. Strid A, Hallström E, Hjorth T, Johansson I, Lindahl B, Sonesson U, Winkvist A, Huseinovic E. Climate impact from diet in relation to background and sociodemographic characteristics in the Västerbotten Intervention Programme. Public health nutrition. 2019 Dec;22(17):3288-97.
363. Strid A, Hallström E, Lindroos AK, Lindahl B, Johansson I, Winkvist A. Adherence to the Swedish dietary guidelines and the impact on mortality and climate in a population-based cohort study. Public Health Nutrition. 2023 Nov;26(11):2333-42.
364. Strid A, Hallström E, Sonesson U, Sjons J, Winkvist A, Bianchi M. Sustainability indicators for foods benefiting climate and health. Sustainability. 2021 Mar 24;13(7):3621.
365. Strid A, Johansson I, Bianchi M, Sonesson U, Hallström E, Lindahl B, Winkvist A. Diets benefiting health and climate relate to longevity in northern Sweden. The American journal of clinical nutrition. 2021 Aug;114(2):515-29.
366. Strid A, Johansson I, Lindahl B, Hallström E, Winkvist A. Toward a More Climate-Sustainable Diet: Possible Deleterious Impacts on Health When Diet Quality Is Ignored. The Journal of Nutrition. 2023 Jan 1;153(1):242-52.
367. Sugimoto M, Murakami K, Sasaki S. Greenhouse gas emissions and energy use of self-selected diet is not associated with diet quality among Japanese adults. Proceedings of the Nutrition Society. 2020;79(OCE2).
368. Sugimoto M, Temme EH, Biesbroek S, Kanellopoulos A, Okubo H, Fujiwara A, Asakura K, Masayasu S, Sasaki S, van't Veer P. Exploring culturally acceptable, nutritious, affordable and low climatic impact diet for Japanese diets: proof of concept of applying a new modelling approach using data envelopment analysis. British Journal of Nutrition. 2022 Dec;128(12):2438-52.
369. Sugimoto, M., Temme, E.H., Biesbroek, S., Kanellopoulos, A., Okubo, H., Fujiwara, A., Asakura, K., Masayasu, S., Sasaki, S. and van't Veer, P., 2022. Exploring culturally acceptable, nutritious, affordable and low climatic impact diet for Japanese diets: proof of concept of applying a new modelling approach using data envelopment analysis. British Journal of Nutrition, pp.1-15.
370. Sun M, Xu X, Hu Y, Ren Y, Zhang L, Wang Y. What differentiates food-related environmental footprints of rural Chinese households?. Resources, Conservation and Recycling. 2021 Mar 1;166:105347.
371. Sundin N, Rosell M, Eriksson M, Jensen C, Bianchi M. The climate impact of excess food intake-An avoidable environmental burden. Resources, Conservation and Recycling. 2021 Nov 1;174:105777.

372. Tainio M, Monsivais P, Jones NR, Brand C, Woodcock J. Mortality, greenhouse gas emissions and consumer cost impacts of combined diet and physical activity scenarios: a health impact assessment study. BMJ open. 2017 Feb 1;7(2):e014199.
373. Tang TW, Sobko T. Environmental Impact of the Average Hong Kong Diet: A Case for Adopting Sustainable Diets in Urban Centers. Challenges. 2019 Dec 6;10(2):5.
374. Telleria-Aramburu N, Bermúdez-Marín N, Rocandio AM, Telletxea S, Basabe N, Rebato E, Arroyo-Izaga M. Nutritional quality and carbon footprint of university students' diets: results from the EHU12/24 study. Public Health Nutrition. 2022 Jan;25(1):183-95.
375. Tello J, Garcillán PP, Ezcurra E. How dietary transition changed land use in Mexico. Ambio. 2020 Oct;49(10):1676-84.
376. Temme EH, Toxopeus IB, Kramer GF, Brosens MC, Drijvers JM, Tyszler M, Ocké MC. Greenhouse gas emission of diets in the Netherlands and associations with food, energy and macronutrient intakes. Public health nutrition. 2015 Sep;18(13):2433-45.
377. Temme EH, van der Voet H, Thissen JT, Verkaik-Kloosterman J, van Donkersgoed G, Nonhebel S. Replacement of meat and dairy by plant-derived foods: estimated effects on land use, iron and SFA intakes in young Dutch adult females. Public Health Nutr. 2013 Oct;16(10):1900-7
378. Tepper S, Kissinger M, Avital K, Shahar DR. The Environmental Footprint Associated With the Mediterranean Diet, EAT-Lancet Diet, and the Sustainable Healthy Diet Index: A Population-Based Study. Frontiers in Nutrition. 2022;9.
379. Terwisscha van Scheltinga C, de Miguel Garcia A, Wilbers GJ, Heesmans H, Dankers R, Smaling E. Unravelling the interplay between water and food systems in arid and semi-arid environments: the case of Egypt. Food Security. 2021 Oct;13(5):1145-61.
380. Tessari P, Lante A, Mosca G. Essential amino acids: master regulators of nutrition and environmental footprint?. Scientific reports. 2016 May 25;6(1):1-3.
381. Thaler S, Zessner M, Weigl M, Rechberger H, Schilling K, Kroiss H. Possible implications of dietary changes on nutrient fluxes, environment and land use in Austria. Agricultural Systems. 2015 Jun 1;136:14-29.
382. Theurl MC, Lauk C, Kalt G, Mayer A, Kaltenegger K, Morais TG, Teixeira RF, Domingos T, Winiwarter W, Erb KH, Haberl H. Food systems in a zero-deforestation world: Dietary change is more important than intensification for climate targets in 2050. Science of the Total Environment. 2020 Sep 15;735:139353.
383. Tom MS, Fischbeck PS, Hendrickson CT. Energy use, blue water footprint, and greenhouse gas emissions for current food consumption patterns and dietary recommendations in the US. Environment Systems and Decisions. 2016 Mar;36(1):92-103.
384. Tompa O, Kanalas O, Kiss A, Soós S, Lakner Z. Integrative analysis of dietary water footprint and dietary quality–Towards the practical application of sustainable nutrition. Acta Alimentaria. 2021 Nov 15;50(4):518-26.
385. Tompa O, Kiss A, Lakner Z. Towards the sustainable food consumption in Central Europe: Stochastic relationship between water footprint and nutrition. Acta Alimentaria. 2020 Mar;49(1):86-92.
386. Tompa O, Kiss A, Maillot M, Sarkadi Nagy E, Temesi Á, Lakner Z. Sustainable Diet Optimization Targeting Dietary Water Footprint Reduction—A Country-Specific Study. Sustainability. 2022 Feb 17;14(4):2309.
387. Tompa O, Lakner Z, Oláh J, Popp J, Kiss A. Is the sustainable choice a healthy choice?—water footprint consequence of changing dietary patterns. Nutrients. 2020 Aug 25;12(9):2578.
388. Topcu B, Dias GM, Mollaei S. Ten-Year Changes in Global Warming Potential of Dietary Patterns Based on Food Consumption in Ontario, Canada. Sustainability. 2022 Jan;14(10):6290.
389. Torstensson L, Johansson R, Mark-Herbert C. Food Dishes for Sustainable Development: A Swedish Food Retail Perspective. Foods. 2021 Apr 23;10(5):932.

390. Treu H, Nordborg M, Cederberg C, Heuer T, Claupein E, Hoffmann H, Berndes G. Carbon footprints and land use of conventional and organic diets in Germany. Journal of Cleaner Production. 2017 Sep 10;161:127-42.
391. Trinh HT, Linderhof V, Vuong VT, Esaryk EE, Heller M, Dijkxhoorn Y, Nguyen TM, Huynh TT, Hernandez R, Duong TT, Luu VT. Diets, food choices and environmental impacts across an urban-rural interface in Northern Vietnam. Agriculture. 2021 Feb 7;11(2):137.
392. Trolle E, Nordman M, Lassen AD, Colley TA, Mogensen L. Carbon Footprint Reduction by Transitioning to a Diet Consistent with the Danish Climate-Friendly Dietary Guidelines: A Comparison of Different Carbon Footprint Databases. Foods. 2022 Jan;11(8):1119.
393. Tseng AA. Equivalent reduction in greenhouse gas emissions by Mahayana Buddhists practicing vegetarian diets. Journal of religion and health. 2020 Feb;59(1):598-613.
394. Tubiello FN, Karl K, Flammini A, Gütschow J, Conchedda G, Pan X, Qi SY, Halldórudóttir Heiðarsdóttir H, Wanner N, Quadrelli R, Rocha Souza L. Pre-and post-production processes increasingly dominate greenhouse gas emissions from agri-food systems. Earth System Science Data. 2022 Apr 14;14(4):1795-809.
395. Tubiello FN, Rosenzweig C, Conchedda G, Karl K, Gütschow J, Xueyao P, Obli-Laryea G, Wanner N, Qiu SY, De Barros J, Flammini A. Greenhouse gas emissions from food systems: building the evidence base. Environmental Research Letters. 2021 Jun 8;16(6):065007.
396. Tulloch AI, Oh RR, Gallegos D. Environmental and public health co-benefits of consumer switches to immunity-supporting food. Ambio. 2022 Jul;51(7):1658-72.
397. Tuninetti M, Ridolfi L, Laio F. Compliance with EAT–Lancet dietary guidelines would reduce global water footprint but increase it for 40% of the world population. Nat Food 3: 143–151.
398. Turner GM, Larsen KA, Candy S, Ogilvy S, Ananthapavan J, Moodie M, James SW, Friel S, Ryan CJ, Lawrence MA. Squandering Australia's food security—The environmental and economic costs of our unhealthy diet and the policy Path We're On. Journal of cleaner production. 2018 Sep 10;195:1581-99.
399. Turner-McGrievy GM, Leach AM, Wilcox S, Frongillo EA. Differences in environmental impact and food expenditures of four different plant-based diets and an omnivorous diet: results of a randomized, controlled intervention. Journal of hunger & environmental nutrition. 2016 Jul 2;11(3):382-95.
400. Tuyishimire A, Liu Y, Yin J, Kou L, Lin S, Lin J, Kubwimana JJ, Moharrami K, Simbi CH. Drivers of the increasing water footprint in Africa: The food consumption perspective. Science of The Total Environment. 2022 Feb 25;809:152196.
401. Tyszler M, Kramer G, Blonk H. Just eating healthier is not enough: studying the environmental impact of different diet scenarios for Dutch women (31–50 years old) by linear programming. The International Journal of Life Cycle Assessment. 2016 May;21(5):701-9.
402. Ulaszewska MM, Luzzani G, Pignatelli S, Capri E. Assessment of diet-related GHG emissions using the environmental hourglass approach for the Mediterranean and new Nordic diets. Science of the Total Environment. 2017 Jan 1;574:829-36.
403. Vallejo RM, Schulz CA, van de Locht K, Oluwagbemigun K, Alexy U, Nöthlings U. Associations Between Adherence to a Dietary Index Based on the EAT-Lancet Reference Diet with Nutritional, Anthropometric and Ecological Sustainability Parameters: Results From the German DONALD Cohort Study. The Journal of Nutrition. 2022 May 12.
404. Van Bussel LM, Kuijsten A, Mars M, Feskens EJ, van't Veer P. Taste profiles of diets high and low in environmental sustainability and health. Food Quality and Preference. 2019 Dec 1;78:103730.
405. Van Bussel LM, Van Rossum CT, Temme EH, Boon PE, Ocké MC. Educational differences in healthy, environmentally sustainable and safe food consumption among adults in the Netherlands. Public health nutrition. 2020 Aug;23(12):2057-67.

406. Van de Kamp ME, Seves SM, Temme EH. Reducing GHG emissions while improving diet quality: exploring the potential of reduced meat, cheese and alcoholic and soft drinks consumption at specific moments during the day. BMC public health. 2018 Dec;18(1):1-2.
407. van de Kamp ME, van Dooren C, Hollander A, Geurts M, Brink EJ, van Rossum C, Biesbroek S, de Valk E, Toxopeus IB, Temme EH. Healthy diets with reduced environmental impact?–The greenhouse gas emissions of various diets adhering to the Dutch food based dietary guidelines. Food Research International. 2018 Feb 1;104:14-24.
408. Van Dooren C, Aiking H. Defining a nutritionally healthy, environmentally friendly, and culturally acceptable Low Lands Diet. The International Journal of Life Cycle Assessment. 2016 May;21(5):688-700.
409. van Dooren C, Douma A, Aiking H, Vellinga P. Proposing a novel index reflecting both climate impact and nutritional impact of food products. Ecological Economics. 2017 Jan 1;131:389-98.
410. Van Dooren C, Tyszler M, Kramer GF, Aiking H. Combining low price, low climate impact and high nutritional value in one shopping basket through diet optimization by linear programming. Sustainability. 2015 Sep 18;7(9):12837-55.
411. Van Mierlo K, Rohmer S, Gerdessen JC. A model for composing meat replacers: Reducing the environmental impact of our food consumption pattern while retaining its nutritional value. Journal of Cleaner Production. 2017 Nov 1;165:930-50.
412. Vanham D, Del Pozo S, Pekcan AG, Keinan-Boker L, Trichopoulou A, Gawlik BM. Water consumption related to different diets in Mediterranean cities. Science of the Total Environment. 2016 Dec 15;573:96-105.
413. Vanham D, Guenther S, Ros-Baró M, Bach-Faig A. Which diet has the lower water footprint in Mediterranean countries?. Resources, Conservation and Recycling. 2021 Aug 1;171:105631.
414. Vanham D, Hoekstra AY, Bidoglio G. Potential water saving through changes in European diets. Environment international. 2013 Nov 1;61:45-56.
415. Vanham D, Mak TN, Gawlik BM. Urban food consumption and associated water resources: The example of Dutch cities. Science of the Total Environment. 2016 Sep 15;565:232-9.
416. Vanham D, Mekonnen MM, Hoekstra AY. The water footprint of the EU for different diets. Ecological indicators. 2013 Sep 1;32:1-8.
417. Vázquez-Rowe I, Larrea-Gallegos G, Villanueva-Rey P, Gilardino A. Climate change mitigation opportunities based on carbon footprint estimates of dietary patterns in Peru. Plos one. 2017 Nov 16;12(11):e0188182.
418. Veeramani A, Dias GM, Kirkpatrick SI. Carbon footprint of dietary patterns in Ontario, Canada: A case study based on actual food consumption. Journal of cleaner production. 2017 Sep 20;162:1398-406.
419. Vellinga RE, van Bakel M, Biesbroek S, Toxopeus IB, de Valk E, Hollander A, van't Veer P, Temme EH. Evaluation of foods, drinks and diets in the Netherlands according to the degree of processing for nutritional quality, environmental impact and food costs. BMC Public Health. 2022 Dec;22(1):1-5.
420. Vellinga RE, van de Kamp M, Toxopeus IB, van Rossum CT, de Valk E, Biesbroek S, Hollander A, Temme EH. Greenhouse gas emissions and blue water use of Dutch diets and its association with health. Sustainability. 2019 Oct 30;11(21):6027.
421. Vellinga RE, van den Boomgaard I, Boer JM, van der Schouw YT, Harbers MC, Verschuren WM, van't Veer P, Temme EH, Biesbroek S. Different Levels of Ultraprocessed Food and Beverage Consumption and Associations with Environmental Sustainability and All-cause Mortality in EPIC-NL. The American Journal of Clinical Nutrition. 2023 May 18.
422. Vetőné Mózner Z. Sustainability and consumption structure: environmental impacts of food consumption clusters. A case study for H ungary. International Journal of Consumer Studies. 2014 Sep;38(5):529-39.
423. Vettera SH, Sapkota TB, Hillier J, Stirling CM, Macdiarmid JI, Aleksandrowicz L, Rosemary Green R, Joye EJ, Dangour AD, Smith P. Greenhouse gas emissions from agricultural food

production to supply Indian diets: implications for climate change mitigation. Agric. Ecosyst. Environ. 2017;237:234-41.
424. Vicente-Vicente JL, Piorr A. Can a shift to regional and organic diets reduce greenhouse gas emissions from the food system? A case study from Qatar. Carbon Balance and Management. 2021 Dec;16(1):1-9.
425. Vidal R, Moliner E, Pikula A, Mena-Nieto A, Ortega A. Comparison of the carbon footprint of different patient diets in a Spanish hospital. Journal of health services research & policy. 2015 Jan;20(1):39-44.
426. Vieux F, Perignon M, Gazan R, Darmon N. Dietary changes needed to improve diet sustainability: are they similar across Europe?. European Journal of Clinical Nutrition. 2018 Jul;72(7):951-60.
427. Vieux F, Soler LG, Touazi D, Darmon N. High nutritional quality is not associated with low greenhouse gas emissions in self-selected diets of French adults. The American journal of clinical nutrition. 2013 Mar 1;97(3):569-83.
428. Vinci G, Maddaloni L, Prencipe SA, Ruggeri M, Di Loreto MV. A Comparison of the Mediterranean Diet and Current Food Patterns in Italy: A Life Cycle Thinking Approach for a Sustainable Consumption. International Journal of Environmental Research and Public Health. 2022 Sep 27;19(19):12274.
429. Vitale M, Giosuè A, Vaccaro O, Riccardi G. Recent trends in dietary habits of the Italian population: Potential impact on health and the environment. Nutrients. 2021 Jan 31;13(2):476.
430. Volanti M, Arfelli F, Neri E, Saliani A, Passarini F, Vassura I, Cristallo G. Environmental Impact of Meals: How Big Is the Carbon Footprint in the School Canteens?. Foods. 2022 Jan 12;11(2):193.
431. von Ow A, Waldvogel T, Nemecek T. Environmental optimization of the Swiss population's diet using domestic production resources. Journal of Cleaner Production. 2020 Mar 1;248:119241.
432. Vora N, Shah A, Bilec MM, Khanna V. Food–energy–water nexus: quantifying embodied energy and GHG emissions from irrigation through virtual water transfers in food trade. ACS Sustainable Chemistry & Engineering. 2017 Mar 6;5(3):2119-28.
433. Walker C, Beretta C, Sanjuán N, Hellweg S. Calculating the energy and water use in food processing and assessing the resulting impacts. The International Journal of Life Cycle Assessment. 2018 Apr;23(4):824-39.
434. Walker C, Gibney ER, Hellweg S. Comparison of environmental impact and nutritional quality among a European sample population–findings from the Food4Me study. Scientific reports. 2018 Feb 5;8(1):1-0.
435. Walker C, Gibney ER, Mathers JC, Hellweg S. Comparing environmental and personal health impacts of individual food choices. Science of The Total Environment. 2019 Oct 1;685:609-20.
436. Walker C, Pfister S, Hellweg S. Methodology and optimization tool for a personalized low environmental impact and healthful diet specific to country and season. Journal of Industrial Ecology. 2021 Oct;25(5):1147-60.
437. Wang L, Cui S, Hu Y, O'Connor P, Gao B, Huang W, Zhang Y, Xu S. The co-benefits for food carbon footprint and overweight and obesity from dietary adjustments in China. Journal of Cleaner Production. 2021 Mar 20;289:125675.
438. Wang L, Gao B, Hu Y, Huang W, Cui S. Environmental effects of sustainability-oriented diet transition in China. Resources, Conservation and Recycling. 2020 Jul 1;158:104802.
439. Wang T, Grech A, Dissanayake HU, Boylan S, Skilton MR. Modeling the Effect of Environmentally Sustainable Food Swaps on Nutrient Intake in Pregnant Women. Nutrients. 2021 Sep 24;13(10):3355.

440. Webb J, Williams AG, Hope E, Evans D, Moorhouse E. Do foods imported into the UK have a greater environmental impact than the same foods produced within the UK?. The International Journal of Life Cycle Assessment. 2013 Aug;18(7):1325-43.
441. Werner LB, Flysjö A, Tholstrup T. Greenhouse gas emissions of realistic dietary choices in Denmark: the carbon footprint and nutritional value of dairy products. Food & nutrition research. 2014 Jan 1;58(1):20687.
442. Westhoek H, Lesschen JP, Rood T, Wagner S, De Marco A, Murphy-Bokern D, Leip A, van Grinsven H, Sutton MA, Oenema O. Food choices, health and environment: Effects of cutting Europe's meat and dairy intake. Global Environmental Change. 2014 May 1;26:196-205.
443. Wickramasinghe K, Rayner M, Goldacre M, Townsend N, Scarborough P. Environmental and nutrition impact of achieving new School Food Plan recommendations in the primary school meals sector in England. BMJ open. 2017 Apr 1;7(4):e013840.
444. Wickramasinghe KK, Rayner M, Goldacre M, Townsend N, Scarborough P. Contribution of healthy and unhealthy primary school meals to greenhouse gas emissions in England: linking nutritional data and greenhouse gas emission data of diets. European journal of clinical nutrition. 2016 Oct;70(10):1162-7.
445. Willits-Smith A, Aranda R, Heller MC, Rose D. Addressing the carbon footprint, healthfulness, and costs of self-selected diets in the USA: a population-based cross-sectional study. The Lancet Planetary Health. 2020 Mar 1;4(3):e98-106.
446. Wilson N, Nghiem N, Ni Mhurchu C, Eyles H, Baker MG, Blakely T. Foods and dietary patterns that are healthy, low-cost, and environmentally sustainable: a case study of optimization modeling for New Zealand. PloS one. 2013 Mar 27;8(3):e59648.
447. Wolfson JA, Willits-Smith AM, Leung CW, Heller MC, Rose D. Cooking at Home, Fast Food, Meat Consumption, and Dietary Carbon Footprint among US Adults. International Journal of Environmental Research and Public Health. 2022 Jan 13;19(2):853.
448. Wrieden W, Halligan J, Goffe L, Barton K, Leinonen I. Sustainable diets in the UK—Developing a systematic framework to assess the environmental impact, cost and nutritional quality of household food purchases. Sustainability. 2019 Sep 11;11(18):4974.
449. Wright EC, van Oort B, Bjøntegaard MM, Carlsen MH, Andersen LF. Environmental and nutritional assessment of young children's diets in Norway: comparing the current diet with national dietary guidelines and the EAT-Lancet reference diet. European Journal of Nutrition. 2023 Dec;62(8):3383-96.
450. Wu H, Wang S, Gao L, Zhang L, Yuan Z, Fan T, Wei K, Huang L. Nutrient-derived environmental impacts in Chinese agriculture during 1978–2015. Journal of environmental management. 2018 Jul 1;217:762-74.
451. Xian CF, Gong C, Lu F, Zhang L, Ouyang ZY. Linking dietary patterns to environmental degradation: the spatiotemporal analysis of rural food nitrogen footprints in China. Frontiers in Nutrition. 2021:588.
452. Xiong X, Zhang L, Hao Y, Zhang P, Chang Y, Liu G. Urban dietary changes and linked carbon footprint in China: a case study of Beijing. Journal of environmental management. 2020 Feb 1;255:109877.
453. Xiong X, Zhang L, Hao Y, Zhang P, Shi Z, Zhang T. How urbanization and ecological conditions affect urban diet-linked GHG emissions: New evidence from China. Resources, Conservation and Recycling. 2022 Jan 1;176:105903.
454. Xu Y, Geng Y, Gao Z, Xiao S, Zhang C, Zhuang M. Accounting greenhouse gas emissions of food consumption between urban and rural residents in China: A whole production perspective. Frontiers in Energy. 2022 Apr;16(2):357-74.
455. Yang X, Chen Q, Chen Q, Xu W, Xu Z. Optimization of Different Carbohydrate-rich Foods Combining Carbon Footprint and Nutritional Value. InIOP Conference Series: Earth and Environmental Science 2019 Oct 1 (Vol. 332, No. 2, p. 022052). IOP Publishing.

456. Yawson DO. Estimating virtual water and land use transfers associated with future food supply: A scalable food balance approach. MethodsX. 2020 Jan 1;7:100811.
457. Ye YX, Geng TT, Zhou YF, He P, Zhang JJ, Liu G, Willett W, Pan A, Koh WP. Adherence to a planetary health diet, environmental impacts, and mortality in Chinese adults. JAMA network open. 2023 Oct 2;6(10):e2339468-.
458. Yin J, Yang D, Zhang X, Zhang Y, Cai T, Hao Y, Cui S, Chen Y. Diet shift: Considering environment, health and food culture. Science of The Total Environment. 2020 Jun 1;719:137484.
459. Yin J, Zhang X, Huang W, Liu L, Zhang Y, Yang D, Hao Y, Chen Y. The potential benefits of dietary shift in China: Synergies among acceptability, health, and environmental sustainability. Science of The Total Environment. 2021 Jul 20;779:146497.
460. Yoo SH, Lee SH, Choi JY, Im JB. Estimation of potential water requirements using water footprint for the target of food self-sufficiency in South Korea. Paddy and water environment. 2016 Jan;14(1):259-69.
461. Yue Q, Xu X, Hillier J, Cheng K, Pan G. Mitigating greenhouse gas emissions in agriculture: From farm production to food consumption. Journal of Cleaner Production. 2017 Apr 15;149:1011-9.
462. Zhang H, Xu Y, Lahr ML. The greenhouse gas footprints of China's food production and consumption (1987–2017). Journal of Environmental Management. 2022 Jan 1;301:113934.
463. Zhang J, Chai L. Trade-off between human health and environmental health in global diets. Resources, Conservation and Recycling. 2022 Jul 1;182:106336.
464. Zhang Y, Tian Q, Hu H, Yu M. Water footprint of food consumption by Chinese residents. International Journal of Environmental Research and Public Health. 2019 Oct;16(20):3979.
465. Zucchinelli M, Spinelli R, Corrado S, Lamastra L. Evaluation of the influence on water consumption and water scarcity of different healthy diet scenarios. Journal of Environmental Management. 2021 Aug 1;291:112687.
466. Zucchinelli M, Sporchia F, Piva M, Thomsen M, Lamastra L, Caro D. Effects of different Danish food consumption patterns on Water ScarcityFootprint. Journal of Environmental Management. 2021 Dec 15;300:113713.
